# Kill Two Birds with One Stone: A Multifunctional Dual‐Targeting Protein Drug to Overcome Imatinib Resistance in Philadelphia Chromosome‐Positive Leukemia

**DOI:** 10.1002/advs.202104850

**Published:** 2022-03-03

**Authors:** Bohan Ma, Hui Feng, Chao Feng, Yi Liu, Hailing Zhang, Jincheng Wang, Wenjuan Wang, Pengcheng He, Fan Niu

**Affiliations:** ^1^ Department of Urology The First Affiliated Hospital Xi'an Jiaotong University Xi'an 710061 China; ^2^ Department of Hematology The First Affiliated Hospital of Xi'an Jiaotong University Xi'an 710061 China; ^3^ Department of Endocrinology The First Affiliated Hospital of Xi'an Jiaotong University Xi'an 710061 China

**Keywords:** Bcr/Abl T315I, Bcr/Abl tetramerization domain, drug resistance, PROTAC, protein drug

## Abstract

The Bcr/Abl plays a central role in Philadelphia chromosome‐positive (Ph+) leukemia because of the constitutively activated Abl tyrosine kinase and its downstream pathways. Currently, the clinical treatment of imatinib‐resistant patients with tyrosine kinase inhibitors is severely limited by drug resistance and adverse effects. Herein, a dual‐targeting proteolysis‐targeting chimera (PROTAC) protein drug, termed ^PMI^Bcr/Abl‐R6, is designed by engrafting an MDM2/p53 inhibition peptide sequence onto the Bcr/Abl tetramerization domain. ^PMI^Bcr/Abl‐R6, harboring a Bcr/Abl targeting sequence and an MDM2 binding sequence, acts as a PROTAC drug in Ph+ leukemia cells. Its dual‐targeting constitution suggests that ^PMI^Bcr/Abl‐R6 designs to target the tetramerization domain instead of the Abl kinase domain, therefore has the potential to overcome drug resistance mutations in the kinase domain. The efficient ability of ^PMI^Bcr/Abl‐R6 is demonstrated to simultaneously induce Bcr/Abl degradation and activate the p53 pathway. ^PMI^Bcr/Abl‐R6 has the potential to overcome drug resistance in Ph+ leukemias by multiple mechanisms.

## Graphical abstract

Imatinib‐resistant Philadelphia chromosome‐positive (Ph+) leukemia treatment remains a challenge. ^PMI^Bcr/Abl‐R6, a designed protein PROTAC (proteolysis‐targeting chimera), targets simultaneously Bcr/Abl tetramerization and p53/MDM2 interaction, both hardly “druggable” by small molecules. ^PMI^Bcr/Abl‐R6 induces Bcr/Abl degradation, despite isoforms and resistance‐related mutations, and activates p53 pathway, providing a different drug design strategy for protein–protein interaction targets.

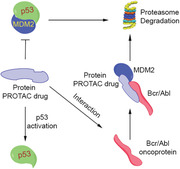

## 1 Introduction

The Philadelphia (Ph) chromosome is an abnormal chromosome resulting from a reciprocal translocation between chromosomes 9 and 22 [t (9; 22].^[^ ^1^ ^]^ This genetic rearrangement gives rise to the fusion protein Bcr/Abl, which plays a central role in the pathogenesis of over 95% of chronic myelogenous leukemia (CML) cases^[^ ^2^ ^]^ and ≈20% of acute lymphoblastic leukemia (ALL) cases.^[^ ^3^ ^]^ Under physiopathological conditions, Bcr/Abl chimeric proteins form tetramers via their oligomerization domain, termed OLI (oligomerization domain), which forcibly gathers Abl kinase domains, thus inducing Abl autophosphorylation/interphosphorylation and subsequent constitutive enzymatic and downstream signaling activation.^[^ ^4^ ^]^ The Abl kinase domain typically represents a traditional drug target, usually including G protein‐coupled receptors, enzymes and ionic channels, which are commonly characterized by the presence of a hydrophobic cavity allowing small‐molecule binding.^[^ ^5^ ^]^ For these reasons, inhibiting Abl kinase activity has attracted much attention in pharmaceutical development in recent decades. Imatinib, the first Abl‐specific tyrosine kinase inhibitor (TKI), competitively binds to its enzymatic activity pocket, has been developed for Ph+ leukemia treatment, and is considered a milestone in targeted cancer therapy.^[^ ^6^ ^]^ However, during clinical application, large numbers of imatinib‐resistant Ph+ leukemia cases have been discovered and reported. There are multiple imatinib‐resistance mechanisms that classically can be divided into two types: Bcr‐Abl‐dependent^[^ ^7^ ^]^ and Bcr‐Abl‐independent mechanisms.^[^ ^8^ ^]^ Bcr‐Abl‐dependent resistance to imatinib is caused by mutations in the Abl kinase enzymatic site that disable TKI binding, especially the mutation T315I (isoleucine replaces threonine at position 315 of Bcr‐Abl), which accounts for 2–20% of CML cases and is still considered incurable.^[^ ^9^ ^]^ On the other hand, Bcr‐Abl‐independent mechanisms consist mainly of increased drug efflux/decreased uptake and the activation of alternative oncopathways.^[^ ^8^ ^]^ Imatinib resistance has promoted the development of new generations of TKIs, such as nilotinib^[^ ^10^ ^]^ and dasatinib^[^ ^11^ ^]^ as the second generation, and bosutinib and ponatinib as the third generation.^[^ ^12^ ^]^ To date, TKIs remain efficacious therapies for most cases of Ph+ leukemias and greatly improve patients’ overall survival. However, new‐generation TKI development must compromise the efficacy and toxicity and have encountered a bottleneck in addressing resistance.^[^ ^13^ ^]^ To overcome this problem, new strategies are necessary to counteract TKI resistance.

The Bcr‐Abl OLI domain is the main structural basis of Bcr‐Abl tetramer formation, making it crucial as a kinase domain for Bcr‐Abl oncogenicity.^[^ ^14^ ^]^ Additionally, although Bcr‐Abl proteins could be classified into three variants p190, p210, p230, depending on the breakpoint of Ph chromosome translocation, the OLI domain is strictly conserved through different variants.^[^ ^15^ ^]^ Therefore, disrupting Bcr‐Abl oligomerization by targeting the OLI domain may provide an alternative therapeutic approach independent of Abl kinase activity. However, the Bcr‐Abl OLI domain occupies a typical protein–protein interface (PPI) and is not very suitable as small‐molecule antagonist target because of the absence of a hydrophobic cavity.^[^ ^14^ ^]^ Peptide drugs, compared with small molecules, often excel due to their high potency and selectivity and low toxicity and are an ideal candidate choice for Bcr‐Abl oligomerization interruption. In fact, previous studies have shown that the Bcr‐Abl oligomerization domain peptide is able to competitively inhibit full‐length Bcr‐Abl oligomerization in a dominant‐negative manner, consequently promoting Ph+ leukemias cell apoptosis, although the efficacy was moderated.^[^ ^16^ ^]^

Recent advances in proteolysis‐targeting chimera (PROTAC) drugs are offering a new opportunity for cancer therapeutic development.^[^ ^17^ ^]^ A PROTAC is a bifunctional molecule composed of one ligand that binds to an E3 ubiquitin ligase and another that binds to the target protein. Instead of competitive inhibition, this molecule can recruit E3 ligase to the protein of interest, subsequently promoting polyubiquitination and removing the target protein by degradation.^[^ ^18^ ^]^ Small‐molecule PROTAC drugs targeting the Bcr/Abl kinase domain have been developed and show better efficacy than imatinib.^[^ ^19^ ^]^ However, PROTAC drug design targeting Bcr/Abl OLI is still lacking.

We analyzed Bcr/Abl OLI (residues 1–72) in more detail and found that residues Bcr_30–65_, which participate in coiled‐coil peptide oligomerization interactions, are sufficient for tetramer formation, while residues 5–15 could serve as an MDM2 binding peptide‐p53/MDM2 inhibitor (PMI, a 12 amino acid peptide) engraft template.^[^ ^20^ ^]^ Therefore, we rationally designed a heterobifunctional protein drug by engrafting the PMI sequence on residues 5–15 of Bcr/Abl. We have proven that ^PMI^Bcr/Abl‐R6 interacts with MDM2 with a *K*_D_ = 49 × 10^−9^ m.^[^ ^21^ ^]^ In this way, MDM2, which can act as an E3 ligase, once forcibly hijacked to bind Bcr/Abl, will promote Bcr/Abl protein degradation.

In Ph+ leukemia cases, to counteract the notably mutation‐free Bcr/Abl‐independent resistance, activating p53 has been proven to be a promising therapeutic approach.^[^ ^22^ ^]^ Rossana and colleagues proved that Bcr/Abl augments MDM2 expression through its downstream pathway, which results in wild‐type p53 inactivation and then dysfunction in Ph+ leukemias.^[^ ^23^ ^]^ Additionally, Abraham et al.^[^ ^24^ ^]^ showed that activating p53 in CML could eliminate cancer stem cells. Therefore, p53 activation through MDM2 could play a synergistic role with Bcr/Abl removal. We demonstrated in our previous publication that the engraft protein drug ^PMI^Bcr/Abl‐R6 could penetrate into the cytoplasm, interact with MDM2 with high affinity, and activate p53‐related apoptosis in *Tp53* wild‐type colon cancer cells in vivo in a xenograft mouse model, showing great stability and low immunogenicity and toxicity both in vitro and in vivo.^[^ ^21^ ^]^

Herein, in part of this work, we report that the engrafted protein drug ^PMI^Bcr/Abl‐R6 is able to achieve interaction between Bcr‐Abl and MDM2‐E3 ligase at the same time and act as a Bcr/Abl‐targeting PROTAC drug, inhibiting the growth of imatinib‐resistant Ph+ leukemia cells and patient‐derived cancer cells, including cells with the T315I mutation. We demonstrate here that ^PMI^Bcr/Abl‐R6 has multiple functions in Ph+ leukemias: interrupting the oligomerization of Bcr/Abl, inducing Bcr/Abl degradation, and activating p53 in p53 wild‐type cancer cells. ^PMI^Bcr/Abl‐R6 could overcome either Bcr/Abl‐dependent or Bcr/Abl‐independent drug resistance.

## 2 Results

### 2.1 Dual‐Functional PROTAC ^PMI^Bcr/Abl‐R6 Peptide Design for Ph+ Leukemia Treatment

**Figure 1:**
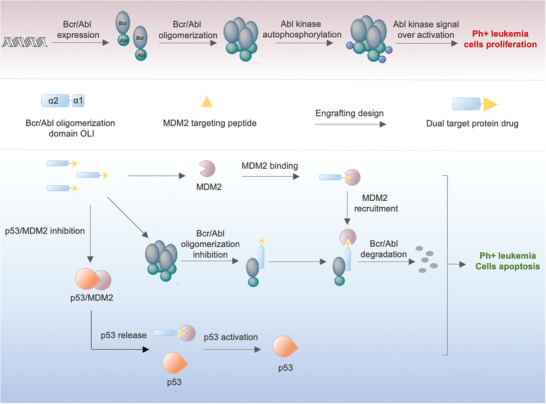
Schematic diagram of ^PMI^Bcr/Abl‐R6 function in Ph+ leukemia cells.

As shown in **Figure** **1**, under physiopathological conditions, Ph+ leukemia cells achieved unlimited cell proliferation by abnormal Abl kinase activity because of Bcr/Abl oligomerization via the OLI domain. According to the structure of the Bcr/Abl tetramer (protein data bank PDB: 1k1y), the Bcr/Abl tetramerization domain is composed of two alpha helices, *α*1 and *α*2. As reported in our previously published work, we engrafted the p53/MDM2 inhibitor peptide sequence‐PMI onto *α*1 and added a 6‐arginine cell penetration sequence, termed ^PMI^Bcr/Abl‐R6. ^PMI^Bcr/Abl‐R6 maintained a tetramer structure and acquired MDM2 binding ability. As PMI targets the p53 binding domain of MDM2, ^PMI^Bcr/Abl‐R6 was proven to competitively release p53 and activate p53 downstream signaling pathways in *Tp53* wild‐type cancer cells.^[^ ^21^ ^]^

In Ph+ leukemia cases, either resistant to TKIs or not, ^PMI^Bcr/Abl‐R6 is thought to be able to kill Ph+ leukemia cells by dual targeting of both, MDM2 and Bcr/Abl (Figure 1). First, ^PMI^Bcr/Abl‐R6 disrupts the oligomerization of the Bcr/Abl fusion protein by interacting with coiled‐coil residues of Bcr/Abl, thus decreasing Abl phosphorylation. Second, ^PMI^Bcr/Abl‐R6 obtained MDM2 binding capacity from the engrafted PMI. In this way, ^PMI^Bcr/Abl‐R6 will interact at the same time with Bcr/Abl and MDM2, which is an E3 ligase. By shortening the spatial distance between MDM2 and Bcr/Abl, ^PMI^Bcr/Abl‐R6 should ultimately induce Bcr/Abl polyubiquitination by MDM2 and Bcr/Abl degradation. Thus, ^PMI^Bcr/Abl‐R6 will act as a PROTAC drug in Ph+ leukemia cells. Finally, ^PMI^Bcr/Abl‐R6 competitively releases p53, which is usually wild type and inactive in leukemias, playing the role of an “MDM2 switch” that changes from suppression of the cancer suppressor p53 to suppression of the oncoprotein Bcr/Abl, efficiently promoting apoptosis via multiple mechanisms.

As ^PMI^Bcr/Abl‐R6 targets Bcr/Abl oligomerization instead of the Abl kinase domain, it will be able to overcome Ph+ leukemia TKI resistance due to Abl mutations, and its multiple mechanisms of cancer cell killing will allow us to reverse drug resistance that is independent of Abl mutations. Accordingly, we obtained the designed protein drug ^PMI^Bcr/Abl‐R6 by solid‐phase peptide synthesis, followed by purification by preparative reversed‐phase HPLC and analysis by analytical reversed‐phase high performance liquid chromatography (HPLC) and electrospray ionization mass spectrometry (ESI‐MS, Figure S1, Supporting Information) to carry out further evaluations.

### 2.2 ^PMI^Bcr/Abl‐R6 Disrupts Oligomerization of Bcr/Abl While Inhibiting p53/MDM2 Interaction and Gains Leukemia Cell‐Penetrating Ability

**Figure 2:**
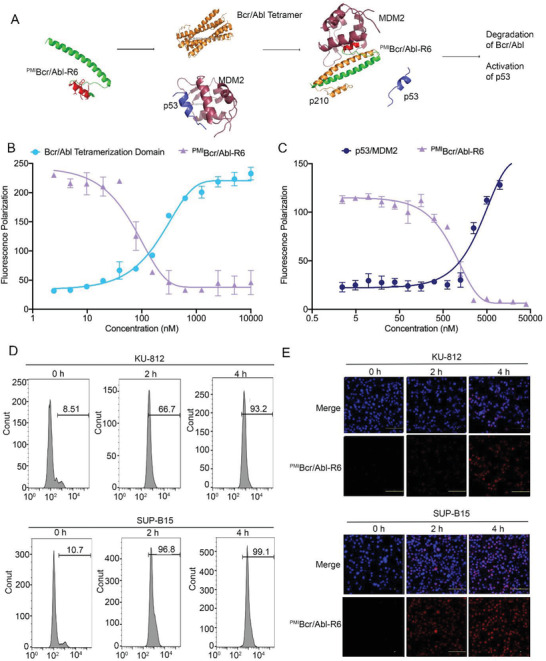
^PMI^Bcr/Abl‐R6 inhibits the oligomerization of Bcr/Abl, inhibits the p53/MDM2 interaction, and the cell penetrating ability. A) ^PMI^Bcr/Abl‐R6 structural and mechanistic scheme. B) Monomer‐tetramer equilibrium of serially diluted Bcr/Abl tetramerization domain (concentrations from 10 × 10^−6^ to 0.3 × 10^−9^ m) detection and the inhibition ability of ^PMI^Bcr/Abl‐R6 for Bcr/Abl oligomerization detection by fluorescence polarization (the Bcr/Abl tetramer was labeled with rhodamine). C) The inhibition ability of ^PMI^Bcr/Abl‐R6 for p53/MDM2 interaction detection by fluorescence polarization (p53 was labeled with rhodamine). D) Flow cytometry analysis of the cell membrane permeability of rhodamine B‐labeled ^PMI^Bcr/Abl‐R6 in KU‐812 and SUP‐B15 cells. E) Confocal microscopy analysis of the cell membrane permeability of rhodamine B‐labeled ^PMI^Bcr/Abl‐R6 in KU‐812 and SUP‐B15 cells. The scale bar represents 100 µm.

^PMI^Bcr/Abl‐R6 functional structure is shown in the scheme in **Figure** **2**A. ^PMI^Bcr/Abl‐R6 interacts with Bcr/Abl on helix *α*2 and interacts with MDM2 on helix *α*1. To determine the oligomerization inhibition ability of ^PMI^Bcr/Abl‐R6, competitive fluorescence polarization (FP) assays were carried out. The Bcr/Abl oligomerization domain sequence was synthesized (Figure S2, Supporting Information) and labeled with rhodamine B (excitation: 535 nm, emission: 580 nm). As shown in Figure 2B, the Bcr/Abl‐rhodamine B FP value increased depending on the Bcr/Abl concentration, meaning that Bcr/Abl forms a tetramer structure. When ^PMI^Bcr/Abl‐R6 was added to the system, the FP value of Bcr/Abl decreased while ^PMI^Bcr/Abl‐R6 concentration increased, indicating that ^PMI^Bcr/Abl‐R6 disrupted Bcr/Abl oligomerization. The maximum FP was taken to indicate 100% tetramerization of Bcr/Abl, and the minimum was taken to indicate the monomer. The FP number could be converted into the tetrameric percentage of the protein and then taken to the fourth power of the concentration as the abscissa and substituted into the protein depolymerization equation. The calculated inhibition constant K*i* of ^PMI^Bcr/Abl‐R6 for Bcr/Abl tetramerization disruption was 150.6 × 10^−9^ m. In parallel, with a similar approach, we determined the binding affinity of ^PMI^Bcr/Abl‐R6 with MDM2. The MDM2 binding domain of p53 (residues 1–107) was synthesized, labeled with rhodamine B and used to determine the binding affinity between p53 and MDM2 (Figure S3, Supporting Information). Then, ^PMI^Bcr/Abl‐R6 was added to the system to evaluate its ability to compete with p53 and bind to MDM2. The maximum FP was taken to correspond to 100% p53/MDM2 complex, and the minimum reflected p53 released from the p53/MDM2 complex. The competitive K*i* of ^PMI^Bcr/Abl‐R6 for the p53/MDM2 complex was 586.5 × 10^−9^ m, as determined in Figure 2C. These data indicate that in vitro, ^PMI^Bcr/Abl‐R6 is able to interrupt Bcr/Abl tetramerization and bind to MDM2, releasing p53.

Peptide drugs usually have high specificity, high affinity, and low toxicity, although their poor stability and lack of membrane penetration greatly limit their application potential. ^PMI^Bcr/Abl‐R6 uses the Bcr/Abl OLI domain as a design template and inherits the excellent stability of the Bcr/Abl OLI domain. This property has been proven in our previous study.^[^ ^21^ ^]^ We also demonstrated that ^PMI^Bcr/Abl‐R6 was able to penetrate the cytoplasm of an epithelial cancer cell line. Here, we further evaluated its cell penetrating ability in leukemia cells by cellular uptake assays using flow cytometry and confocal microscopy analysis. We labeled ^PMI^Bcr/Abl‐R6 with rhodamine B and chose two Ph+ leukemia cell lines: KU‐812 and SUP‐B15. As shown in Figure 2D, flow cytometry analysis proved that ^PMI^Bcr/Abl‐R6 entered KU‐812 and SUP‐B15 in a time‐dependent manner. After 4 h of incubation, ^PMI^Bcr/Abl‐R6 entered almost all leukemia cells, especially ^PMI^Bcr/Abl‐R6 SUP‐B15. Confocal microscopy analysis confirmed the results of flow cytometry (Figure 2E).

### 2.3 ^PMI^Bcr/Abl‐R6 Kills Imatinib‐Resistant Ph+ CML and ALL Leukemia Cells

**Figure 3:**
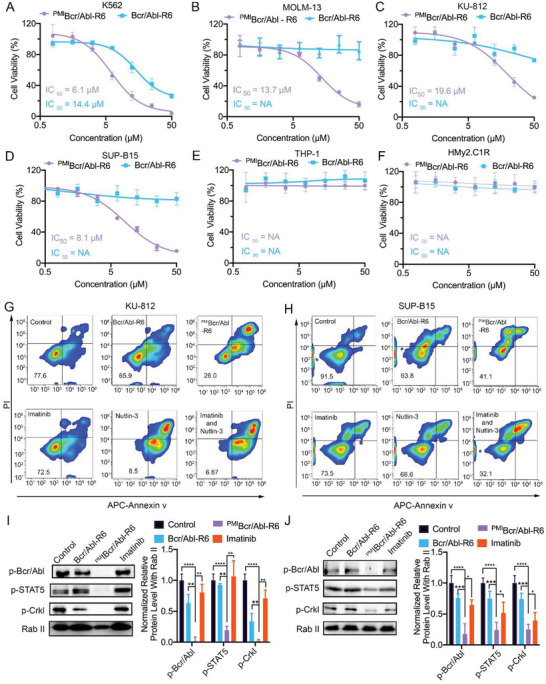
^PMI^Bcr/Abl‐R6 kills Ph+ leukemia cells in vitro. Cell viability of A) K562, B) MOLM‐13, C) KU‐812, D) SUP‐B15, E) THP‐1, F) and HMy2.C1R cells 48 h after treatment with varying concentrations of Bcr/Abl‐R6 and PMIBcr/Abl‐R6. Representative data on apoptosis of G) KU‐812 and H) SUP‐B15 24 h after treatment with Bcr/Abl‐R6 (10 × 10^−6^ m ), ^PMI^Bcr/Abl‐R6 (10 × 10^−6^ m), imatinib (1 × 10^−6^ m ), nutlin‐3 (10 × 10^−6^ m), and cotreatment of imatinib and nutlin‐3 as analyzed by flow cytometry. Representative western blotting and statistical analysis of p‐Bcr/Abl, p‐STAT5, and p‐Crkl in I) KU‐812 and J) SUP‐B15 cells after treatment with Bcr/Abl‐R6, ^PMI^Bcr/Abl‐R6, and imatinib. (Statistical analysis was carried out based on data from three independent assays, *n* = 3; ** indicates *p* < 0.01; ***indicates *p* < 0.001.)

We evaluated the function of ^PMI^Bcr/Abl‐R6 in inhibiting leukemia cells. First, we tested the efficacy of ^PMI^Bcr/Abl‐R6 on single‐target leukemia cell lines to separately validate its inhibitory role. As shown in **Figure** **3**A, we treated K562 cells, which are a Ph+ and *Tp53*‐deleted CML cell line, with different concentrations of ^PMI^Bcr/Abl‐R6 and Bcr/Abl‐R6. The results showed that both the engrafted drug ^PMI^Bcr/Abl‐R6 and the oligomerization domain sequence of Bcr/ABl‐R6 alone inhibited K562 cell viability. However, the IC_50_ of ^PMI^Bcr/Abl‐R6 is almost half that of Bcr/ABl‐R6, proving that inhibiting Bcr/Abl oligomerization shows efficacy in treating Ph+ leukemia cells, our PROTAC drug functions more effectively than the original antagonist. ^PMI^Bcr/Abl‐R6, playing the role of a PROTAC drug, could remove Bcr/Abl oncoprotein by inducing its degradation at protein level, while Bcr/Abl‐R6 only inhibits Bcr/Abl tetramerization by competing their interaction. And numerous studies also showed PROTAC drugs are more efficient than the original antagonist.^[^ ^25^ ^]^ MOLM‐13 is an acute myeloid leukemia cell line that overexpresses wild‐type p53 and MDM2 but lacks the Philadelphia chromosome. ^PMI^Bcr/Abl‐R6 effectively inhibited the growth of MOLM‐13 cells, as expected, while Bcr/Abl‐R6 did not show any toxicity (Figure 3B).

To evaluate the synergistic functions of ^PMI^Bcr/Abl‐R6, we chose two imatinib‐resistant cell lines, KU‐812 (CML) and SUP‐B15 (ALL), which both bear the Philadelphia chromosome, the wild‐type *Tp53* gene and overexpressed MDM2.^[^ ^26^ ^]^ As shown in Figure 2C,D, ^PMI^Bcr/Abl‐R6 showed great killing efficacy in both KU‐812 and SUP‐B15 cells. And as shown in Figure S4 in the Supporting Information, imatinib efficiently inhibits cell viability in K562 cells, while show moderate efficacy in KU‐812 and SUP‐B15 cells. The half‐maximal inhibitory concentration (IC_50_) values of ^PMI^Bcr/Abl‐R6 were 6.1 × 10^−6^ and 13.7 × 10^−6^ m, respectively. In contrast, the nonengrafted peptide drug Bcr/Abl‐R6 did not efficiently inhibit cell viability in the concentration range tested. Finally, to evaluate the toxicity of ^PMI^Bcr/Abl‐R6, we chose two Ph‐negative cell lines, THP‐1 and Hmy.2C1R (Figure 3E,F). THP‐1 is an acute myeloid leukemia cell line bearing *Tp53* deletion and the absence of the Ph chromosome. HMy.2C1R is an immortalized normal lymphocyte B cell line. Neither ^PMI^Bcr/Abl‐R6 nor Bcr/Abl‐R6 showed any nonspecific toxicity to either THP‐1 cells or Hmy.2C1R cells (Figure 3E,F) at the range of concentrations tested. All these data proved that the rationally designed peptide drug ^PMI^Bcr/Abl‐R6 was able to inhibit Ph+ leukemia cells, MDM2 targeting improved its efficacy, and no off‐target toxicity was observed.

The mechanism of ^PMI^Bcr/Abl‐R6 was then further evaluated. KU‐812 and SUP‐B15 cells were treated with ^PMI^Bcr/Abl‐R6 (10 × 10^−6^ m), Bcr/Abl‐R6 (10 × 10^−6^ m), imatinib (1 × 10^−6^ m), or nutlin‐3 (10 × 10^−6^ m) for 24 h. These concentrations corresponded to their IC_50_ values in K562 cells. Apoptosis assay data show (Figure 3G,H, statistical tests showed in Figures S5 and S6, Supporting Information) that ^PMI^Bcr/Abl‐R6 effectively induced apoptosis in the imatinib‐resistant cell lines KU‐812 and SUP‐B15. Moreover, in SUP‐B15 cells, the combination of imatinib and nutin‐3 also showed similar efficacy. Although in KU‐812, nutlin‐3 alone could effectively induce cellular apoptosis.

To investigate whether ^PMI^Bcr/Abl‐R6 inhibits the Bcr/Abl signaling pathway in KU‐812 and SUP‐B15, KU‐812, and SUP‐B15 cells were treated with ^PMI^Bcr/Abl‐R6 (10 × 10^−6^ m), Bcr/Abl‐R6 (10 × 10^−6^ m), imatinib (1 × 10^−6^ m), or nutlin‐3 (10 × 10^−6^ m) for 24 h. Immunoblotting analysis of phosphorylated Bcr/Abl, phosphorylated STAT5 and phosphorylated Crkl was performed, and Rab11 was used as a loading control. As shown in Figure 3I,J, ^PMI^Bcr/Abl‐R6 inhibited Bcr/Abl phosphorylation and its downstream signaling pathway in KU‐812 and SUP‐B15 cells, while Bcr/Abl‐R6 and imatinib showed nonsignificant or only moderate efficacy at the used concentrations.

### 2.4 ^PMI^Bcr/Abl‐R6 Induces Bcr/Abl p210 and p190 Degradation and Activates p53 in Ph+ Leukemia

**Figure 4:**
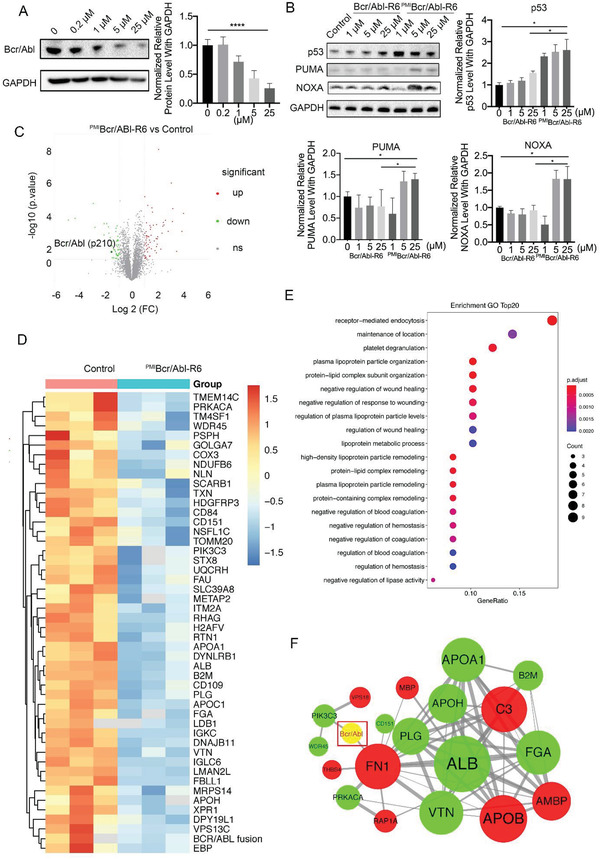
^PMI^Bcr/Abl‐R6 induces Bcr/Abl degradation, activates p53 and affects the Bcr/Abl and p53 pathways in KU‐812 cells. A) Representative western blotting of Bcr/Abl (p210) after treatment with PMIBcr/Abl‐R6 at different concentrations and statistical analysis of data from three independent assays (*n* = 3; * indicates *p* < 0.05, **** indicates *p* < 0.0001). B) Representative western blotting analysis of p53, PUMA, and NOXA in KU‐812 cells after 24 h of treatment with Bcr/Abl‐R6 (10 × 10^−6^ m ), ^PMI^Bcr/Abl‐R6 (10 × 10^−6^ m ), or imatinib (1 × 10^−6^ m) and the statistical analysis of data from three independent experiments (*n* = 3). C) Volcano plots of proteomics analysis in KU‐812 cells after treatment with ^PMI^Bcr/Abl‐R6 at a concentration of 10 × 10^−6^ m for 24 h. D) Heatmap analysis of proteomics analysis in KU‐812 cells after treatment with ^PMI^Bcr/Abl‐R6 (10 × 10^−6^ m ) for 24 h compared to vehicle treated control group. E) GO pathway enrichment analysis of downregulated proteins after ^PMI^Bcr/Abl‐R6 treatment compared to control group in KU‐812 cells. F) Protein–protein interactions related to Bcr/Abl analysis in KU‐812 cells after treatment with ^PMI^Bcr/Abl‐R6.

To evaluate the degradation ability of ^PMI^Bcr/Abl‐R6 for the Bcr/Abl p210 variant, KU‐812 cells, bearing Bcr/Abl p210 and wild‐type p53, were treated with different concentrations of ^PMI^Bcr/Abl‐R6 for 24 h, and then immunoblotting analysis of Bcr/Abl p210 was performed. The immunoblotting results of p210 showed that ^PMI^Bcr/Abl‐R6 reduced the accumulation of Bcr/Abl in a dose‐dependent manner (**Figure** **4**A). Moreover, immunoblotting analysis of the p53 signaling pathway proved that ^PMI^Bcr/Abl‐R6 efficiently increased p53 protein levels and activated p53 pathway proteins such as p53 up‐regulated modulator of apoptosis (PUMA) and phorbol‐12‐myristate‐13‐acetate‐induced protein 1 (NOXA) (Figure 4B) in a dose‐dependent manner. These data showed that ^PMI^Bcr/Abl‐R6 is able to simultaneously induce Bcr/Abl p210 degradation and p53 activation. Meanwhile, we evaluated Bcr/Abl mRNA level through RT‐PCR in K562, KU‐812, and SUP‐B15 cells after a 24 h treatment with ^PMI^Bcr/Abl‐R6 at different concentrations, (as shown in Figure S7, Supporting Information), data showed that the Bcr/Abl messenger RNA (mRNA) level was not significantly affected, indicating that ^PMI^Bcr/Abl‐R6 reduces Bcr/abl oncoprotein level but not by reducing its transcription.

To accurately evaluate the degradation ability of ^PMI^Bcr/Abl‐R6, label‐free proteomics analysis was performed after ^PMI^Bcr/Abl‐R6 and Bcr/Abl‐R6 treatment at 10 × 10^−6^ m for 24 h. A total of 236 657 spectra were obtained, in which 30 050 peptides were mapped in the database to identify proteins. A total of 4703 proteins were identified. Furthermore, the ^PMI^Bcr/Abl‐R6‐treated group was analyzed compared to the control group and to the Bcr/Abl‐R6‐treated group, and a fold change greater than 2 was used as the selection criterion. As shown in Figure 4C, ^PMI^Bcr/Abl‐R6 treatment caused the downregulation of 51 proteins and the accumulation of 63 compared to the vehicle‐treated control group. The expression patterns of downregulated and upregulated proteins are shown in heatmaps in Figure 4D and Figure S8A in the Supporting Information, respectively. In particular, proteomics analysis proved that ^PMI^Bcr/Abl‐R6 effectively reduced Bcr/Abl protein levels compared to the control group (Figure 4C,D), as well as to the template Bcr/Abl‐R6‐treated group (Figure S9A,B, Supporting Information). However, a similar analysis showed that the template Bcr/Abl‐R6 did not significantly reduce the abundance of Bcr/Abl compared to either control group (Figure S10A,B, Supporting Information). Based on these selected proteins, the Gene Ontology (GO) term distribution of biological processes, cellular components, and molecular functions was also analyzed for ^PMI^Bcr/Abl‐R6 downregulated and upregulated patterns, and the top 20 are presented in Figure 4E and Figure S8B in the Supporting Information, respectively. Interestingly, selected proteins were enriched in lipoprotein metabolism process, platelet degranulation and coagulation process (Figure 4E), and the ubiquitin ligase, peptidase activity and palmitoyl‐protein hydrolase related activity processes (Figure S8B, Supporting Information), which were reported to be closely related to Bcr/Abl p210 CML. Moreover, the protein–protein interaction (PPI) networks of Bcr/Abl are shown in Figure 4F. The PPI of ^PMI^Bcr/Abl‐R6‐induced protein variation showed that fibronectin 1 (FN1) and PIK3C3 interacted directly with Bcr/Abl, indicating that these proteins may be important for the ^PMI^Bcr/Abl‐R6 mechanism.

**Figure 5:**
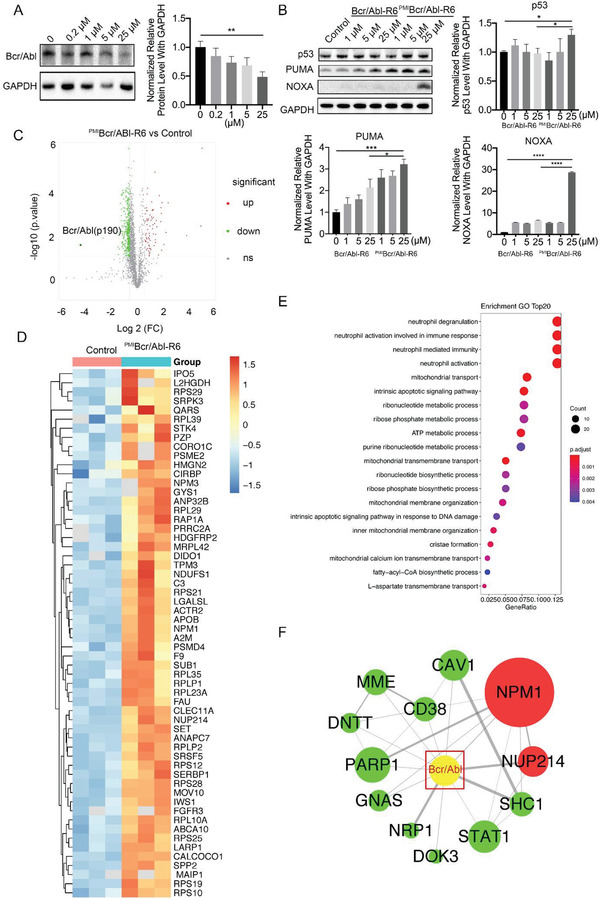
^PMI^Bcr/Abl‐R6 induces Bcr/Abl degradation, activates p53, and affects the Bcr/Abl and p53 pathways in SUP‐B15 cells. A) Representative western blotting and analysis of Bcr/Abl (p190) after treatment with ^PMI^Bcr/Abl‐R6 at different concentrations and statistical analysis of data from three independent assays (*n* = 3; * indicates *p* < 0.05, ** indicates *p* < 0.005; *** indicates *p* < 0.001, **** indicates *p* < 0.0001.) B) Representative western blotting analysis of p53, PUMA, and NOXA in SUP‐B15 cells after 24 h of treatment with Bcr/Abl‐R6 (10 × 10^−6^ m ), ^PMI^Bcr/Abl‐R6 (10 × 10^−6^ m ), or imatinib (1 × 10^−6^ m) and statistical analysis of data from three independent experiments (*n* = 3). C) Volcano plots of proteomics analysis in SUP‐B15 cells after treatment with ^PMI^Bcr/Abl‐R6 at a concentration of 10 × 10^−6^ m for 24 h. D) Heatmap proteomics analysis of upregulated proteins in SUP‐B15 cells after treatment with ^PMI^Bcr/Abl‐R6 compared to vehicle treated control group. E) GO pathway enrichment analysis of downregulated proteins after ^PMI^Bcr/Abl‐R6 treatment compared to control group in SUP‐B15 cells. F) Protein–protein interactions related to Bcr/Abl analysis in SUP‐B15 cells after treatment with ^PMI^Bcr/Abl‐R6.

Similarly, in the Bcr/Abl p190 and wilt‐type p53 phenotype ALL cell line SUP‐B15, which was treated with ^PMI^Bcr/Abl‐R6 for 24 h, immunoblotting analysis of p190 showed that ^PMI^Bcr/Abl‐R6 induced the degradation of Bcr/Abl in a dose‐dependent manner (**Figure** **5**A). Moreover, immunoblotting analysis of the p53 signaling pathway proved that ^PMI^Bcr/Abl‐R6 efficiently activated the p53 pathway in this imatinib‐resistant ALL cell line (Figure 5B). A label‐free proteomics analysis was also performed after ^PMI^Bcr/Abl‐R6 and Bcr/Abl‐R6 treatment at 10 × 10^−6^ m for 24 h in SUP‐B15 cells. A total of 126 154 spectra were obtained, of which 15 162 peptides were mapped in the database to identify proteins. A total of 2671 proteins were identified. Furthermore, similar analysis of data was carried out with identical criteria to KU‐812 cells. ^PMI^Bcr/Abl‐R6 treatment induced the downregulation of 211 proteins and the accumulation of 72 compared to the vehicle‐treated control group (Figure 5C). The expression patterns of downregulated and upregulated proteins are shown in heatmaps in Figures S5D and S11A in the Supporting Information, respectively. In particular, proteomics analysis also proved that ^PMI^Bcr/Abl‐R6 effectively reduced Bcr/Abl protein levels compared to the control group (Figure 5C and Figure S11A, Supporting Information), and to the Bcr/Abl‐R6‐treated group (Figure S12A,B, Supporting Information). However, with a similar analysis, the template protein Bcr/Abl‐R6 did not significantly reduce the abundance of Bcr/Abl compared to either control group (Figure S13A,B, Supporting Information). Based on these selected proteins, the GO term distributions of biological processes, cellular components, and molecular functions for upregulated and downregulated was also analyzed separately, among which the top 20 were presented in Figure 5E and Figure S11B in the Supporting Information, respectively. Interestingly, selected proteins were enriched in neutrophil immunity response‐related processes, ribonucleotide metabolism, apoptosis processes, fatty acid metabolism (Figure 5E), mRNA metabolism, and endoplasmic reticulum‐related processes (Figure S11B, Supporting Information), which were reported to be closely related to Bcr/Abl p190 ALL. Moreover, the PPI networks of Bcr/Abl in this case are shown in Figure 5F. The PPI of ^PMI^Bcr/Abl‐R6 treatment‐induced protein variation showed that SHC1, NUP214, NPM1, CAV1, CD38, MME, DNTT, PARP1, GNAS, NRP1, DOK3, and STAT1 interacted directly with Bcr/Abl, indicating that these proteins may be important for the ^PMI^Bcr/Abl‐R6 mechanism in p190 ALL.

Interestingly, ^PMI^Bcr/ABl‐R6 showed different extent of Bcr/Abl degradation and p53 accumulation. As KU‐812 and SUP‐B15 bear different Bcr/Abl isoforms, Bcr/Abl p210 consists of two more domains—Dbl‐homology (DH) and Pleckstrin‐homology (PH) domain in protein structure than p190. DH domain acts as a guanine nucleotide exchange factor, whereas the PH domain binds to various phosphatidylinositol‐phosphates. PH‐domain mutants alter subcellular localization and result in decreased interactions with p210‐selective interaction partners.^[^ ^27^ ^]^ Also, p210 and p190 differentially activate signaling pathways, such as engage with molecules that indicate interaction with different subcellular compartments. P210 has an increased engagement of molecules active proximal to the membrane, while p190 has an increased engagement of molecules of the cytoskeleton.^[^ ^28^ ^]^ In plus, disease background of KU‐812 and SUP‐B15 is different. This is the possible reason that ^PMI^Bcr/Abl‐R6 did not show identical effect in KU‐812 and SUP‐B15 cells. The proteomics analysis of KU‐812 and SUP‐B15 support the differences between Ph+ CML and Ph+ ALL.

The combination of ^PMI^Bcr/Abl‐R6 efficiently kills imatinib‐resistant both CML and ALL cell lines, and we proved that ^PMI^Bcr/Abl‐R6 could induce different Bcr/Abl isoforms regarding the Abl kinase domain structure.

### 2.5 ^PMI^Bcr/Abl‐R6 Induces Bcr/Abl p185 and Exogenous Expression Bcr/Abl Bearing Drug Resistance Mutations

**Figure 6:**
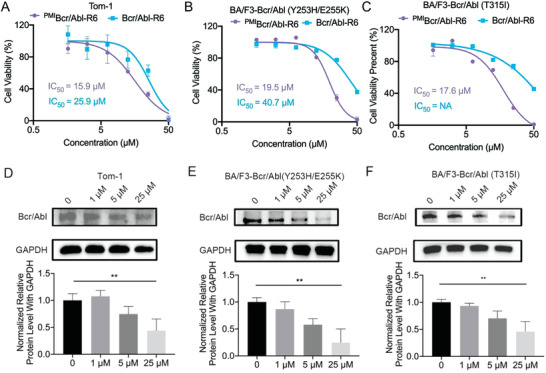
^PMI^Bcr/Abl‐R6 induces Bcr/Abl (p185) and exogenous Bcr/Abl (bearing drug resistance mutations) degradation. Cell viability of A) Tom‐1, B) BA/F3‐Bcr/Abl (Y253H/E255K), and C) BA/F3‐Bcr/Abl (T315I) cells 48 h after treatment with varying concentrations of Bcr/Abl‐R6 and ^PMI^Bcr/Abl‐R6. D) Representative western blotting of Bcr/Abl (p185) in Tom‐1 cells after treatment with ^PMI^Bcr/Abl‐R6 at different concentrations and statistical analysis of data from three independent assays (*n* = 3; **indicates *p* < 0.005). E) Representative western blotting of Bcr/Abl (Y235H/E255K) in BA/F3‐Bcr/Abl (Y253H/E255K) cells after treatment with ^PMI^Bcr/Abl‐R6 at different concentrations and statistical analysis of data from three independent assays (*n* = 3; **indicates *p* < 0.005). F) Representative western blotting of Bcr/Abl (T315I) in BA/F3‐Bcr/Abl (T315I) after treatment with ^PMI^Bcr/Abl‐R6 at different concentrations and statistical analysis of data from three independent assays (*n* = 3; **indicates *p* < 0.005).

In order to investigate efficiency of ^PMI^Bcr/Abl‐R6 in Bcr/Abl p185 variant positive cells and in cells bearing drug resistance‐ related Bcr/Abl mutations. Cell viability tests following imatinib treatment confirmed their resistance (Figure S14, Supporting Information). We tested killing ability and degradation ability of ^PMI^Bcr/Abl‐R6 in Tom‐1 cells, BA/F3‐Bcr/Abl (Y253H/E255K) cells, and BA/F3‐Bcr/Abl (T315I) cells, which are all p53 wild type. As shown in **Figure** **6**A–C, ^PMI^Bcr/Abl‐R6 showed efficacy in inhibiting the cell viability in Tom‐1, murine interleukin‐3 dependent pro‐B cell line BA/F3‐Bcr/Abl (Y253H/E255K), and BA/F3‐Bcr/Abl (T315I) cells, with IC_50_ of 15.9, 19.5, and 17.6 µM, respectively. Although Bcr/Abl‐R6 showed less efficient in these three cell lines. Then Tom‐1, BA/F3‐Bcr/Abl (Y253H/E255K), and BA/F3‐Bcr/Abl (T315I) cells were treated with ^PMI^Bcr/Abl‐R6 at different concentrations for 24 h, and immunoblotting analysis of Bcr/Abl was performed. The immunoblotting results showed that ^PMI^Bcr/Abl‐R6 reduced Bcr/Abl (p185), Bcr/Abl (p210 Y253H/E255K), and Bcr/Abl (p210 T315I) in a dose‐dependent manner (Figure 6D–F). Moreover, immunoblotting analysis of the p53 signaling pathway proved that ^PMI^Bcr/Abl‐R6 efficiently increased p53 protein levels in Tom‐1 cells (Figure S15, Supporting Information).

### 2.6 ^PMI^Bcr/Abl‐R6 Induces Apoptosis of Primary CML and ALL Cells Ex Vivo

**Figure 7:**
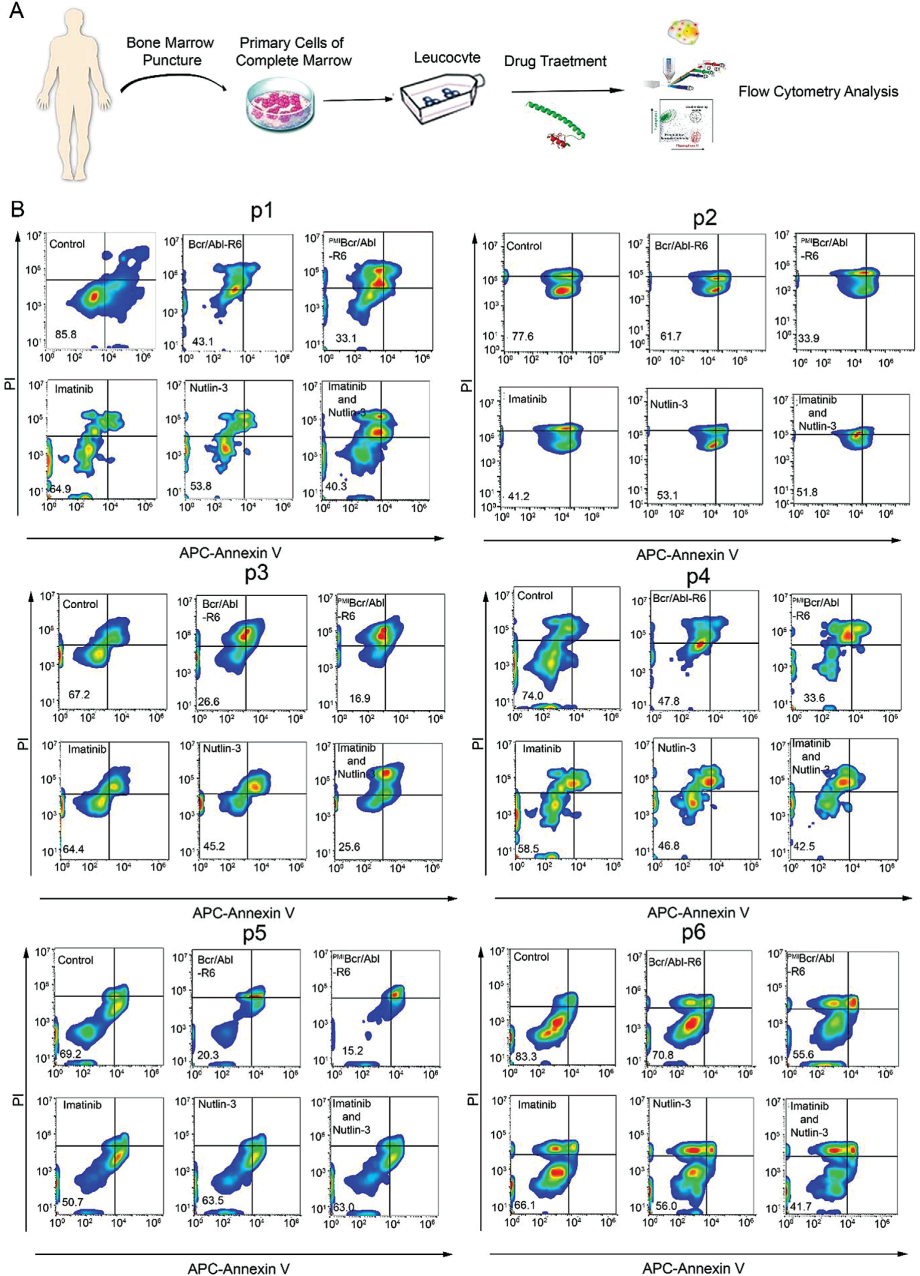
The ex vivo sensitivity of primary cells from patients with CML to Bcr/Abl‐R6 and ^PMI^Bcr/Abl‐R6. A) Schematic of the treatment for leukemia patient specimens. B) Apoptosis of CML specimens after ^PMI^Bcr/Abl‐R6, Bcr/Abl‐R6, imatinib, or nutlin‐3 treatment detected by flow cytometry.

**Figure 8:**
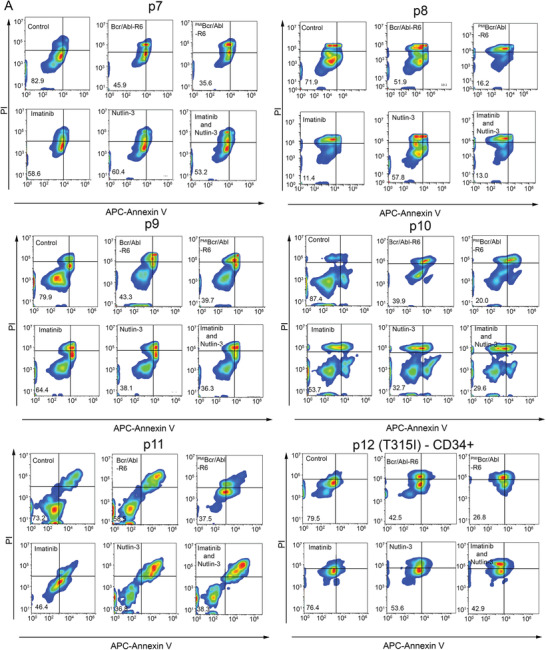
The ex vivo sensitivity of primary cells from patients with B‐ALL to Bcr/Abl‐R6 and ^PMI^Bcr/Abl‐R6. Apoptosis of B‐ALL specimens after ^PMI^Bcr/Abl‐R6, Bcr/Abl‐R6, imatinib, or nutlin‐3 treatment detected by flow cytometry.

Ex vivo cytotoxicity assays using isolated cancer cells from patients are a powerful tool for evaluating the therapeutic efficacy of antitumor agents against leukemias. We obtained primary leukemic cells from both Ph+ CML and ALL patients, of which the baseline information, such as Bcr/Abl variant type, drug resistance, and mutations including *Tp53* mutations, is presented in **Table** **1**, and treated them with ^PMI^Bcr/Abl‐R6, Bcr/Abl‐R6, imatinib, or nutlin‐3 (**Figure** **7**A). We obtained primary leukemic cells from 10 CML (Figure 7B and Figure S16A, Supporting Information) and 6 ALL (**Figure** **8**) patients. As shown in Figure 7B and Figures S8 and S16A, in the Supporting Information, ^PMI^Bcr/Abl‐R6 showed highly efficient apoptosis induction in both Bcr/Abl‐positive CML and ALL. Compared with the template drug Bcr/Abl‐R6, ^PMI^Bcr/Abl‐R6 showed greater potency at the same concentration. Unsurprisingly, combination drug therapy of imatinib and nutlin‐3 showed a certain potential to overcome drug resistance in ALL, indicating that p53 could be considered a drug target in imatinib‐resistant Ph+ leukemias. In particular, sample leukemia stem cells from patient 12 (Figure 8), who had imatinib‐resistant ALL bearing multiple mutations, including T315I, were isolated by CD34‐positive sorting and treated with a single dose of ^PMI^Bcr/Abl‐R6 for 24 h. The results showed that ^PMI^Bcr/Abl‐R6 was able to induce apoptosis, despite their imatinib resistance. We also performed immunoblotting assays of Bcr/Abl and p53 in four ex vivo Ph+ leukemia samples (p13–16). The results, as shown in Figure S16B–E in the Supporting Information, indicated that in all of these four ex vivo samples, ^PMI^Bcr/Abl‐R6 was able to simultaneously induce Bcr/Abl protein degradation and p53 activation.

**Table 1:** Patients’ information

Patient number	Gender	Age	Diagnosis	Bcr/Abl variant	Drug resistance and mutations (including *Tp53*)
P1	Female	47	CML	p210	No
P2	Male	71	CML	p210	No
P3	Male	45	CML	p210	No
P4	Male	49	CML	p210	No
P5	Male	62	CML	p210	No
P6	Female	53	CML	p210	No
P7	Male	62	B‐ALL/LBL	p190	No
P8	Male	50	B‐ALL	p210	No
P9	Female	57	B‐ALL	p190	IKZF1
P10	Female	59	B‐ALL	p210	No
P11	Female	37	B‐ALL	p190	No
P12	Female	49	B‐ALL	p190	Bcr/Abl (Y253H, E255K/V, T315I)
P13	Female	76	CML	NA	No
					
P14	Female	12	CML	p210	No
P15	Male	21	CML	p210	CBF*β*‐MYH11
P16	Male	44	CML	p210	No

### 2.7 ^PMI^Bcr/Abl‐R6 Inhibits Tumor Growth in BA/F3‐Bcr/Abl (T315I) Xenograft Models In Vivo

**Figure 9:**
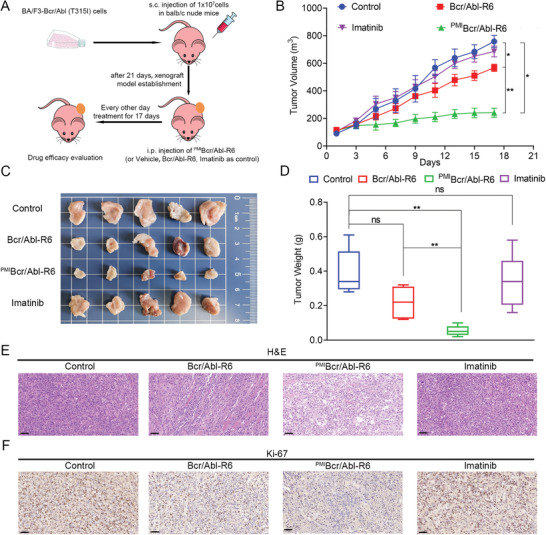
The ^PMI^Bcr/Abl‐R6 effectively inhibits BA/F3‐Bcr/Abl (T315I) tumor growth in vivo. A) Schematic diagram of BA/F3‐Bcr/Abl (T315I) xenograft tumor model establishment and drug treatment. Twenty balb/c nude mice bearing BA/F3‐Bcr/Abl (T315I) tumors, subcutaneously established for three weeks at a palpable volume (around 100 mm^3^), were randomly divided into four groups (*n* = 5), and then treated every other day for 17 d via i.p. injection of 20 × 10^−3^ m Tris‐HCl (control), Bcr/Abl‐R6, ^PMI^Bcr/Abl‐R6, or imatinib at a dose of 5 mg Kg^−1^. B) Tumor growth curves of BA/F3‐Bcr/Abl (T315I) xenografts in balb/c nude mice treated as indicated. The data are presented as the mean ± standard error of mean (SEM) values (*n* = 5), statistical analysis was performed by *t*‐test; ** stands for *p* < 0.01; * for *p* < 0.05. C) Photos of BA/F3‐Bcr/Abl (T315I) xenograft tumors excised at the end of the experiment after different drug treatments. D) Average weight of tumors excised from each group of mice at the end of drug treatment. The data are presented as the mean ± SD values (*n* = 5), statistical analysis was performed using *t*‐test; ** indicates *p* < 0.01; ns for not statistically significant. E) Histopathological analysis of the excised tumors from each treatment group using H&E staining assay (scale bar: 50 µm). H) IHC analysis of Ki‐67 in BA/F3‐Bcr/Abl (T315I) xenograft tumors from each treatment group (scale bar: 50 µm).

To evaluate the therapeutic efficacy of ^PMI^Bcr/Abl‐R6 in vivo, we established subcutaneous xenograft mouse models with BA/F3‐Bcr/Abl (T315I) cells. Twenty tumor‐bearing mice were randomly divided into four groups and treated every other day with 20 × 10^−3^ m Tris‐HCl (control) and Bcr/Abl‐R6, ^PMI^Bcr/Abl‐R6 or imatinib at a dose of 5 mg Kg^−1^ by intraperitoneal injection (**Figure** **9**A). As shown in Figure 9B–D, ^PMI^Bcr/Abl‐R6 potently inhibit BA/F3‐Bcr/Abl (T315I) tumors growth, while Bcr/Abl‐R6 only showed basal ability of tumor inhibition, imatinib as negative control showed no efficiency on BA/F3‐Bcr/Abl (T315I) tumors. Consistent with the detection from the in vivo efficacy study, histopathological analysis using hematoxylin and eosin (H&E) (Figure 9E) staining techniques revealed massive necrotic tumor cells in the tumor tissues from the ^PMI^Bcr/Abl‐R6‐treated group. Immunohistochemical (IHC) analysis of Ki‐67 (Figure 9F and Figure S17, Supporting Information) in each treatment group also proved that ^PMI^Bcr/Abl‐R6 effectively inhibited BA/F3‐Bcr/Abl (T315I) cells growth in vivo. To evaluate safety of ^PMI^Bcr/Abl‐R6, animal body weight variation of different treatment group was monitored. As shown in Figure S18 in the Supporting Information, ^PMI^Bcr/Abl‐R6 did not affect mice body weight. Histopathological analysis of organs at the end of drug treatment using H&E staining (Figure S19, Supporting Information) did not show any toxicity of ^PMI^Bcr/Abl‐R6.

To detective plasma protein binding ability of ^PMI^Bcr/Abl‐R6, HPLC analysis was performed to detect residual ^PMI^Bcr/Abl‐R6 after incubating with fetal bovine serum (FBS) as shown in Figure S20 in the Supporting Information. There are more than 90% residual ^PMI^Bcr/Abl‐R6 detected after overnight incubation and dialysis with 10% and 30% FBS. Even in 50% FBS incubation, there were over 50% of ^PMI^Bcr/Abl‐R6 remains. The results indicate that ^PMI^Bcr/Abl‐R6 show great circulation ability in blood system.

These data indicated that in the real world, the designed multifunctional PROTAC ^PMI^Bcr/Abl‐R6 peptide should have great potency for Ph+ leukemia treatment, including various forms of imatinib resistance, even that of T315I.

## 3 Discussion

Ph+ leukemias represent an important subset of clinical leukemias. Despite the increase in overall survival by imatinib and second‐generation TKIs, drug resistance in Ph+ CML still threatens the patient's life, causing poor clinical outcomes.^[^ ^29^ ^]^ Most resistances of CML rely on Bcr/Abl amino acid substitutions, mainly within the kinase domain.^[^ ^30^ ^]^ One of the most frequent mutations, ranging from 2% to 20% of CML cases, is T315I.^[^ ^31^ ^]^ T315I is regarded as a “gatekeeper” mutation and is the deadliest case since it leads to resistance to second‐generation TKIs, such as nilotinib and dasatinib.^[^ ^32^ ^]^ In Ph+ ALL and CML blasts, TKIs, including nilotinib and dasatinib, showed poor efficiency.^[^ ^33^ ^]^ Developing new therapeutic approaches and novel drugs for drug‐resistant Ph+ leukemias remains a challenge.

Here, taking a different approach, we report a novel PROTAC targeting the oligomerization domain to bypass mutations in the Bcr/Abl kinase domain. Compared with small molecular PROTAC drug, peptide PROTAC drug possesses larger interface of interaction thus usually have a higher affinity and higher specificity for smooth protein interface, especially for *α* helix structure.^[^ ^34^ ^]^ For protein–protein interaction interface, peptide PROTAC composed of more advantages than small molecular PROTAC drugs.^[^ ^35^ ^] PMI^Bcr/Abl‐R6 interacts with the Bcr/Abl oligomerization domain and has a high affinity for the E3 ligase MDM2. In this way, ^PMI^Bcr/Abl‐R6 could effectively induce degradation of both wild‐type Bcr/Abl (p210 and p190) and Bcr/Ablkinase mutants, including but not limited to T315I. In particular, ^PMI^Bcr/Abl‐R6 could activate p53 at the same time. In our previous publication, we evaluated the ^PMI^Bcr/Abl‐R6‐activated p53 signaling pathway in colon cancer in vitro and in vivo. Here, we proved that in Ph+ leukemia cells, ^PMI^Bcr/Abl‐R6 effectively activated p53 in cancer cells with wild‐type p53 and induced apoptosis of Ph+ leukemia cells. The protein p53 has attracted the most attention, as a high proportion of leukemias retain wild‐type p53. Additionally, activating p53 eliminates CML stem cells.^[^ ^24^ ^]^ In summary, ^PMI^Bcr/Abl‐R6 functions by two pathways: Bcr/Abl and p53. We have demonstrated the dual function of ^PMI^Bcr/Abl‐R6 in Ph+ leukemia cells. ^PMI^Bcr/Abl‐R6 revealed great efficacy in cancer cells isolated from patients, including an ALL patient bearing Y253H, E255K/V, and T315I mutations. Our data indicate that the Bcr/Abl pathway and the p53 pathway could play synergistic roles as targets of drug combinations. By proteomics analysis in KU‐812 and SUP‐B15 cells after ^PMI^Bcr/Abl‐R6 treatment, ^PMI^Bcr/Abl‐R6 showed the effects of different signaling pathways. In p210‐positive CML, ^PMI^Bcr/Abl‐R6 showed a greater effect on the lipoprotein metabolism process, platelet degranulation and coagulation process. In p190‐positive ALL, ^PMI^Bcr/ABl‐R6 showed a greater effect on mRNA metabolism and endoplasmic reticulum‐related processes. The different effects of ^PMI^Bcr/Abl‐R6 on the signaling pathway in KU‐812 and SUP‐B15 cells indicate that the roles of p210 and p190 are quite different. Therefore, ^PMI^Bcr/Abl‐R6 also provided another tool for studying the differences between p210 and p190 signaling pathways.

Peptide drug development is usually limited because of poor stability and a lack of cell membrane penetration ability.^[^ ^36^ ^]^ However, ^PMI^Bcr/Abl‐R6 is a complete protein drug without introducing any other components, such as nanodelivery systems. In our previous publication, we evaluated the immunogenicity and biodistribution of ^PMI^Bcr/Abl‐R6. As an intracellularly targeted protein drug, at the same in vivo dose of 5 mg kg^−1^, ^PMI^Bcr/Abl‐R6 showed better efficacy than a small molecular drug (nutlin‐3).^[^ ^21^ ^]^ Intracellularly targeted protein drug design remains a challenge because of limited druggability. Our design of ^PMI^Bcr/Abl‐R6 provides another strategy taking advantage of humanized protein scaffolds.

Herein, we report that the novel dual‐target PROTAC protein drug ^PMI^Bcr/Abl‐R6 is capable of killing imatinib – resistant Ph+ leukemia cells by targeting the Bcr/Abl oligomerization domain, inducing Bcr/Abl degradation and activating the p53 pathway. For Bcr/Abl‐positive leukemia therapy, we provide another brand‐new solution.

## 4 Experimental Section

### Peptide Synthesis

Boc amino acids were obtained from the Peptides Institute (Japan). *p*‐Methyl‐benzhydrylamine(BHA) resin and Boc‐Leu‐OCH2−4‐(oxomethyl)‐phenylacetamidomethyl (PAM) resin were purchased from Applied Biosystems (Foster City, CA, USA). Tris‐(2‐carboxyethyl) phosphine, glutathione, dichloromethane, *N*,*N*‐dimethylformamide, HPLC‐grade acetonitrile, triisopropylsilane, *N*,*N*‐diisopropylethylamine (DIEA), p‐cresol, and ultrapure guanidine hydrochloride (GuHCl) were obtained from Sigma‐Aldrich (St. Louis, MO, USA) and trifluoroacetic acid was purchased from Halocarbon (River Edge, NJ, USA).

All peptides were synthesized on appropriate resin using the optimized O‐Benzotriazole‐N,N,N',N'‐tetramethyl‐uronium‐hexafluorophosphate (HBTU) activation/DIEA in situ neutralization protocol developed by Kent and colleagues for Boc‐chemistry solid phase peptide synthesis on a Csbio CS336X synthesizer. After cleavage and deprotection in hydrofluoric acid (HF), the crude products were precipitated with cold ether and purified to homogeneity by preparative C18 reversed‐phase HPLC. The molecular masses were ascertained by ESI‐MS.

### Fluorescence Marking of Proteins

Proteins were dissolved at a concentration of 1 mg mL^−1^ in PBS pH 7.2. N‐hydroxysuccinimide (NHS)‐activated rhodamine B was then added at a molar ratio to the peptide drug of 2:1 and incubated overnight at room temperature. Excess free rhodamine was removed by performing dialysis three times against phosphate buffer saline (PBS) buffer.

### Fluorescence Polarization

FP can be used to detect the oligomerization state of proteins and protein–protein interactions. When Bcr/Abl‐R6 forms tetramers, the increase in protein size can lead to an increase in the FP value. First, Bcr/Abl‐R6 was labeled with the fluorophore‐rhodamine B (excitation: 535 nm, emission: 580 nm). The maximum FP is regarded as 100% tetramerization of the protein and the minimum is regarded as the monomer. The FP number could be converted into the tetrameric percentage of the protein, taken to the fourth power of the concentration as the abscissa, and substituted into the protein depolymerization equation. For p53 and MDM2 interaction, p53 was labeled with rhodamine B. The maximum FP was regarded as 100% p53/MDM2 complex and the minimum as p53 released from the p53/MDM2 complex.

FP experiments were performed in Microfluor 96‐well plates (Thermo Fisher Scientific) using a Tecan Ultra plate reader. To detect the interaction between ^PMI^Bcr/Abl‐R6 and the Bcr/Abl tetramerization domain, the concentration of rhodamine labeled with the Bcr/Abl tetramerization domain was reduced from 10 000 × 10^−9^ to 2 × 10^−9^ m at equal dilutions. To detect the interaction between ^PMI^Bcr/Abl‐R6 and MDM2, the p53/MDM2 complex concentration was reduced from 50 000 × 10^−9^ to 5 × 10^−9^ m by equal dilution steps.

### Cell Culture

K562, KU812, SUP‐B15, and MOLM‐13 cell lines were purchased from German Collection of Microorganisms and Cell Cultures GmbH (DMSZ). THP‐1, Tom‐1, and Hmy.2CIR cells were obtained from American Tissue Culture Colection (ATCC). BA/F3‐Bcr/Abl (Y253H/E255K) cells and BA/F3‐Bcr/Abl (T315I) cells were purchased from Cobioer. K562, MOLM‐13, THP‐1, Hmy.2CIR, BA/F3‐Bcr/Abl (Y253H/E255K), and BA/F3‐Bcr/Abl (T315I) cells were cultured in RPMI (Roswell Park Memorial Institute)1640 medium (Gibco) containing 10% FBS (Gibco). KU812 and SUP‐B15 cell lines were cultured in Iscove's modified Dulbecco's medium supplemented with 10% FBS (Gibco). All these cells were cultured in suspension at a density of 3 × 10^5^ to 2 × 10^6^ cells mL^−1^.

### Cell Viability Tests by Cell Counting Kit‐8 (CCK‐8)

Cell viability was evaluated by the CCK‐8 assay. Leukemia cells were plated at a density of 3 × 10^5^ cells mL^−1^ on 96‐well plates and three independent samples were seeded in triplicate for each concentration. Cell viability tests were performed 48 h after treatment with Bcr/Abl‐R6 or ^PMI^Bcr/Abl‐R6. After a 2 h incubation with CCK‐8 reagents, the absorbance values at 450 nm were determined using a microplate reader. The CCK‐8 kit was purchased from Dojindo Laboratories.

### Apoptosis Assay by Flow Cytometry

The ability of ^PMI^Bcr/Abl to promote apoptosis was evaluated by flow cytometry using an apoptosis detection kit (Biolegend) including an allophycocyanin (APC)‐labeled anti‐annexin V antibody and a propidium iodide solution.

Cells were seeded at a density of 3 × 10^5^ cells mL^−1^ in 12‐well plates and treated with ^PMI^Bcr/Abl at 10 × 10^−6^ m, while a nontreated control and positive controls treated with imatinib (1 × 10^−6^ m), nutlin‐3 (10 × 10^−6^ m), and imatinib combined with nutlin‐3 were established in parallel. After 24 h of incubation, cells were collected and analyzed by flow cytometry with the mentioned apoptosis kit, rigorously following the instructions.

### Immunoblotting Analysis

Immunoblotting was performed to determine the Bcr/Abl‐ and p53‐related signaling pathway proteins, including phosphorylated Abl (p‐Abl), p‐STAT5, p‐Crkl, p53, PUMA, NOXA, glyceraldehyde‐3‐phosphate dehydrogenase (GAPDH), and Rab 11 as internal controls. Cells were treated with Bcr/Abl‐R6 or ^PMI^Bcr/Abl‐R6 at different concentrations for 48 h. Cellular lysates were collected and the protein concentrations were quantified by a Pierce bicinchoninic acid (BCA) Protein Assay Kit (Thermo Fisher). Equal amounts of total proteins were loaded for western blotting analysis. Primary antibodies were purchased from Santa Cruz Biotechnology (DO‐1 p53), Calbiochem (PUMA and Noxa), Sigma‐Aldrich (GAPDH), and Cell Signaling Technology, Inc (CST: Bcr; p‐Abl, pSTAT‐5, p‐Crkl, Rab 11 cocktail). Secondary antibodies conjugated with horseradish peroxidase were purchased from Calbiochem. Protein expression from immunoblotting was quantified by ImageJ software, and *t*‐tests were used for the statistical analysis of three independent assays for p‐Abl, p‐Crkl, and p‐STAT5. Analysis of variance (ANOVA) tests were used to analyze data on Bcr/Abl degradation and p53 stabilization from three independent experiments.

### Proteomic Analysis

KU‐812 and SUP‐B15 cells were seeded at 3 × 10^5^ cells mL^−1^, followed by treatment with Bcr/Abl‐R6 or ^PMI^Bcr/Abl‐R6 for 24 h. Finally, cells were collected and washed three times with PBS, and label‐free quantitative proteomics and bioinformatics analyses were performed by Bioprofile in Shanghai. Three independent tests were carried out for each group. The data were analyzed and plotted using R software. When selecting protein accumulation or reduction, log2(FC)> = 1 and *p* < 0.05 were chosen as the criteria. The GO term enrichment analysis was performed through the “cluster profiler” and “org.Hs.eg.db” R packages. The protein–protein interaction network was analyzed using STRING (https://cn.string‐db.org). The differentially expressed proteins were uploaded to the STRING database to construct the interaction network of differentially expressed proteins. Then the completed protein–protein interactive network is imported into Cytoscape software for visualization. The nodes in the figure are proteins and the edges are interactions. The size of a node in an interactive network is directly proportional to the degree of interactions, meaning the more edges connected with the node, larger the node. The color of the node is related to the differential expression of the protein represented, red stands for upregulated proteins and green for downregulated proteins.

### Bcr/Abl mRNA Level Detection by RT‐PCR

K562, KU‐812, and SUP‐B15 cells were seeded at 3 × 10^5^ cells mL^−1^, treated with ^PMI^Bcr/Abl‐R6 at different concentrations for 24 h. Cells were then collected by centrifuge and total mRNA was extracted using RNA extraction kit (purchased from Fastagen) following instructors’ procedure. RNA integrity was determined by Nanodrop. cDNA was obtained using primescript RT master mix (purchased from Takara). Bcr/Abl mRNA quantified by reverse transcription‐polymerase chain reaction (RT‐PCR) using Bio‐rad iTaq Univer Synergy Brands (SYBR) Biorad with primers listed as following.

Bcr/Abl mRNA level were presented as relative to *β*‐actin, based on calculations of 2(−*σσ*Ct). The primer sequences for target genes were listed as following.^[^ ^37^ ^]^

*β*‐actin:
Forward: TACCTCATGAAGATCCTCACC
Reverse: TTTCGTGGATGCCACAGGAC
Bcr/Abl p210:
Forward: ENF501: TCCGCTGACCATCAATAAGGA
Reverse: ENR561: CACTCAGACCCTGAGGCTCAA
Bcr/Able p190:
Forward: ENF402: CTGGCCCAACGATGGCGA
Reverse: ENR561: CACTCAGACCCTGAGGCTCAA

Statistical significance was defined as *p* < 0.05 as measured by the t test using GraphPad Prism 10 software (GraphPad, San Diego, CA, USA).

### Sorting of Patient Bone Marrow Aspiration Samples

CML and ALL patients’ bone marrow samples were used for the functional testing of ^PMI^Bcr/Abl. To isolate monocytes from the collected bone marrow, 6% hydroxyethyl starch was added to the samples at 1:1 and incubated for 45 min. Centrifugation for 10 min at 1500 rpm allowed the separation of red cells from monocytes. The upper fraction, which contained monocytes, was collected and washed three times with PBS. To remove the remaining red cells, 4x red blood cell lysis buffer was added. After 5 min of incubation, the monocytes were recovered by centrifugation at 1500 rpm for 10 min. Finally, the cell pellets were cultured in RPMI‐1640 medium with 10% FBS at a density of 3 × 10^6^ cells mL^−1^.

CD34+ cells were sorted using a magnetic microbead‐conjugated CD34 antibody (Miltenyi Biotec) and adequate columns and magnetic separators (MACS), rigorously following the manufacturer's instructions.

All experiments on bone marrow and blood samples from patients performed were under the supervision of the Ethics Committee of the First Affiliated Hospital of Xi'an Jiaotong University (2020 G‐100).

### Plasma Protein Binding Detection of ^PMI^Bcr/Abl‐R6

^PMI^Bcr/Abl‐R6 was dissolved and refolded in PBS with concentration of 1 mg kg^−1^. Then ^PMI^Bcr/Abl‐R6 was separately incubated with PBS, 10% FBS, 30% FBS, and 50% FBS. Residual ^PMI^Bcr/Abl‐R6 were dialyzed into PBS with 25 KD dialysis tubes followed detection by HPLC.

### Antitumor Activity Test in BA/F3‐Bcr/Abl (T315I) Xenograft Model

All experimental procedures involving animals were conducted in accordance with Institution Guidelines and were approved by the Laboratory Animal Center of Xi'an Jiaotong University.

1 × 10^7^ of BA/F3‐Bcr/Abl T315I cells were mixed with matrigel and were subcutaneously injected into balb/c nude mice. After three weeks, the subcutaneous model was established when the tumor volume achieved around 100 mm^3^. Treatment of four groups of mice (five mice per group) was initiated (i.e., day 1) after the tumor had been established after 3 weeks as a palpable mass (around 100 mm^3^ in size). Four medication groups received a 17 d treatment regimen with i.p. every other day. ^PMI^Bcr/Abl‐R6, Bcr/Abl‐R6 and imatinib was administered at dose of 5 mg kg^−1^. Tumor length and width were measured with calipers and tumor volume was calculated using the following equation: tumor volume (*V*) = length × width^2^/2. All IHC staining images were scored using ImageJ.

### Statistical Analysis

Data are expressed as the mean ± standard deviation (SD). Statistical significance between the values was assessed using Student's *t*‐test and two‐way ANOVA via Tukey and Dunnett's multiple compared tests. The proteomic data were analyzed using R software (version 4.1.0). When selecting protein accumulation or reduction, log2(FC)> = 1 and *p* < 0.05 were chosen as the criteria. The GO term enrichment analysis was performed through the “cluster profiler” and “org.Hs.eg.db” R packages. The protein–protein interaction network was analyzed using STRING (https://cn.string‐db.org/). All analysis for statistically significant differences were calculated via GraphPad Prism 8.0. Statistical significance was represented by *p < 0.05, **p < 0.005, ***p < 0.001, and ****p < 0.0001.

## Conflict of Interest

The authors declare no conflict of interest.

## Supporting information

Supporting Information

## Data Availability

The data that support the findings of this study are available in the Supporting Information of this article.
