# Supplementary material for: Kill Two Birds with One Stone: A Multifunctional Dual‐Targeting Protein Drug to Overcome Imatinib Resistance in Philadelphia Chromosome‐Positive Leukemia
Source: Adv Sci (Weinh). 2022 Mar 3;9(13):2104850. doi: 10.1002/advs.202104850 (PMC9069375; doi:10.1002/advs.202104850)
Supplement: Supplementary file 1 — Supporting Information [file ADVS-9-2104850-s001.pdf]

ADVANCED SCIENCE
Open Access

Supporting Information

for *Adv. Sci.*, DOI 10.1002/advs.202104850

Kill Two Birds with One Stone: A Multifunctional Dual-Targeting Protein Drug to Overcome Imatinib Resistance in Philadelphia Chromosome-Positive Leukemia

Bohan Ma, Hui Feng, Chao Feng, Yi Liu, Hailing Zhang, Jincheng Wang, Wenjuan Wang, Pengcheng He and Fan Niu**

Supplemental Information

Kill Two Birds with One Stone: A Multifunctional Dual-Targeting Protein Drug to Overcome Imatinib Resistance in Philadelphia Chromosome-Positive Leukemia

Bohan Ma[A†], Hui Feng[B†], Chao Feng[C], Yi Liu[B], Hailing Zhang[B], Jincheng Wang[B], Wenjuan Wang[B], Pengcheng He[B], Fan Niu[B]**

[A] Department of Urology, The First Affiliated Hospital, Xi’an Jiaotong University, Xi’an, China

[B] Department of Hematology, The First Affiliated Hospital of Xi’an Jiaotong University, Xi’an, China

[C] Department of Endocrinology, The First Affiliated Hospital of Xi'an Jiaotong University, Xi'an, China

†These authors contributed equally to this work.

* Corresponding authors

E-mail : niufan@xjtufh.edu.cn; hepengcheng@xjtu.edu.cn

Keywords: Bcr/Abl Tetramerization Domain, Drug Resistance, Bcr/Abl T315I, PROTAC, Protein Drug

Supplementary Figures

Figure S1

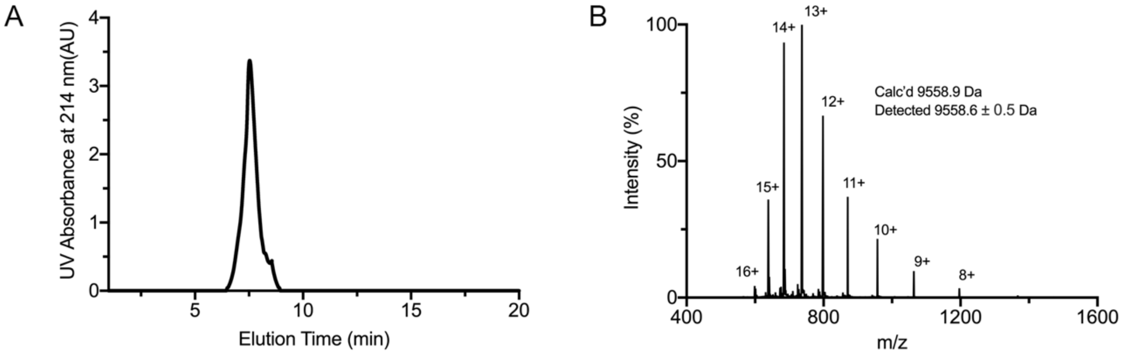

Figure S1. Purification and characterization of synthesized ^{PMI}Bcr/Abl-R6.

Figure S2

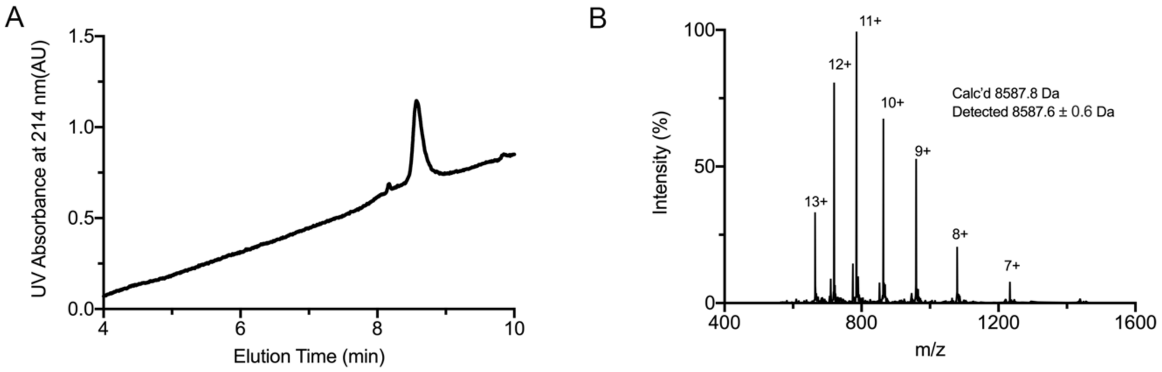

Figure S2. Purification and characterization of synthesized Bcr/Abl tetramerization domain.

Figure S3

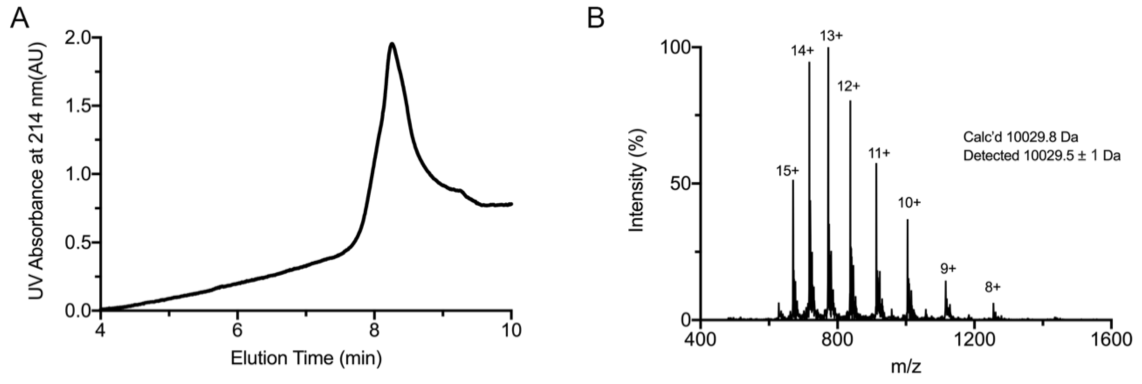

Figure S3. Purification and characterization of synthesized MDM2 N-terminal domain.

Figure S4

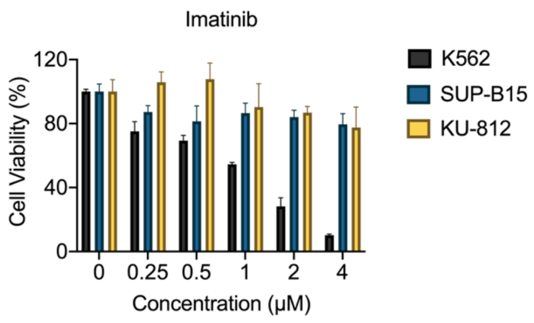

Figure S4. Cell viability of K562, KU-812 and SUP-B15 cells 24h after treatment with varying concentrations of imatinib.

Figure S5

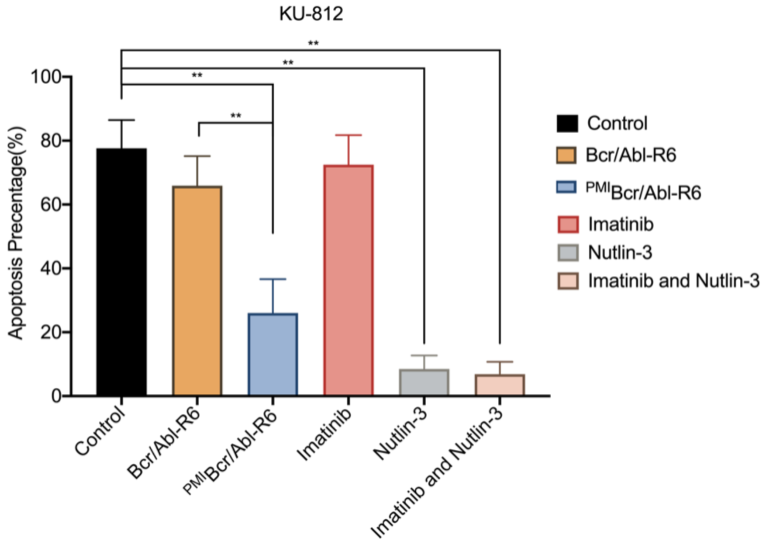

Figure S5. Statistical analysis of apoptosis of KU-812 cells quantified by flow cytometry. Annexin V positive percentage and/or PI positive cells percentage were pooled and considered as apoptotic. Three independent FACS assays were performed, and data are shown as the means ± SD (n = 3). p values were calculated by t-test.

Figure S6

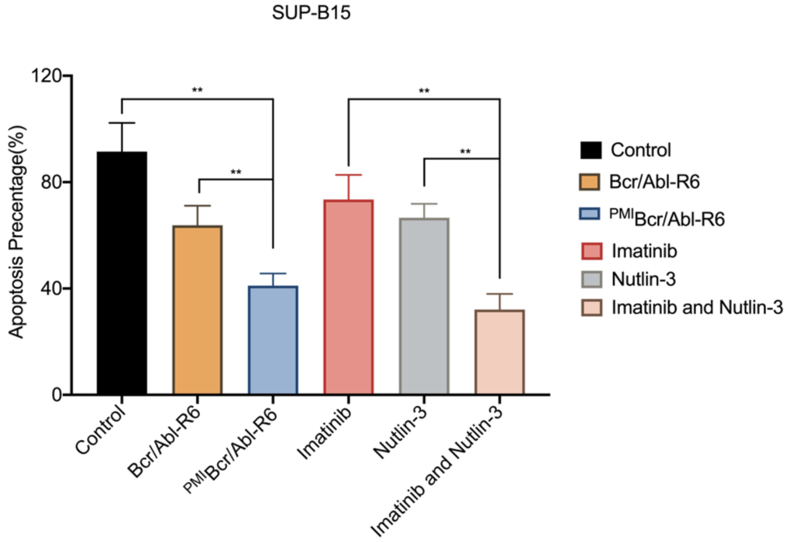

Figure S6. Statistical analysis of apoptosis of SUP-B15 cells quantified by flow cytometry. Annexin V positive percentage and/or PI positive cells percentage were pooled and considered as apoptotic. Three independent FACS assays were performed, and data are shown as the means ± SD (n = 3). p values were calculated by t-test.

Figure S7

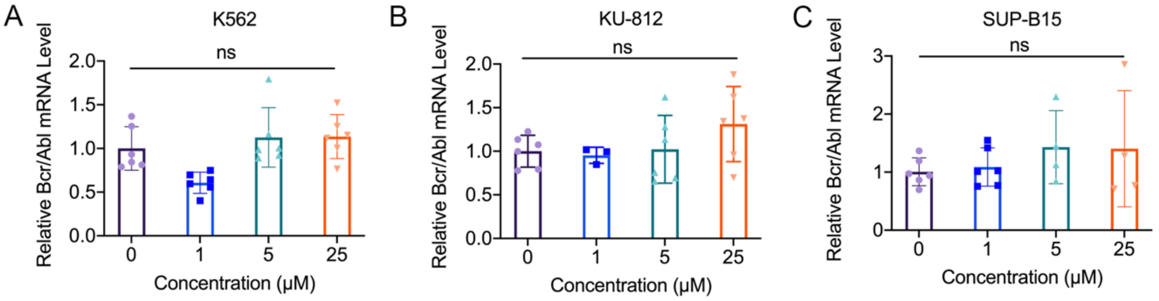

Figure S7. ^{PMI}Bcr/Abl-R6 didn't reduce Bcr/Abl mRNA level in K562, KU-812 or SUP-B15 cells. (A) ^{PMI}Bcr/Abl-R6 does not reduce mRNA level of AR in K562 cells. (B) ^{PMI}Bcr/Abl-R6 does not reduce mRNA level of AR in KU-812 cells. (C) ^{PMI}Bcr/Abl-R6 does not reduce mRNA level of AR in SUP-B15 cells.

Figure S8

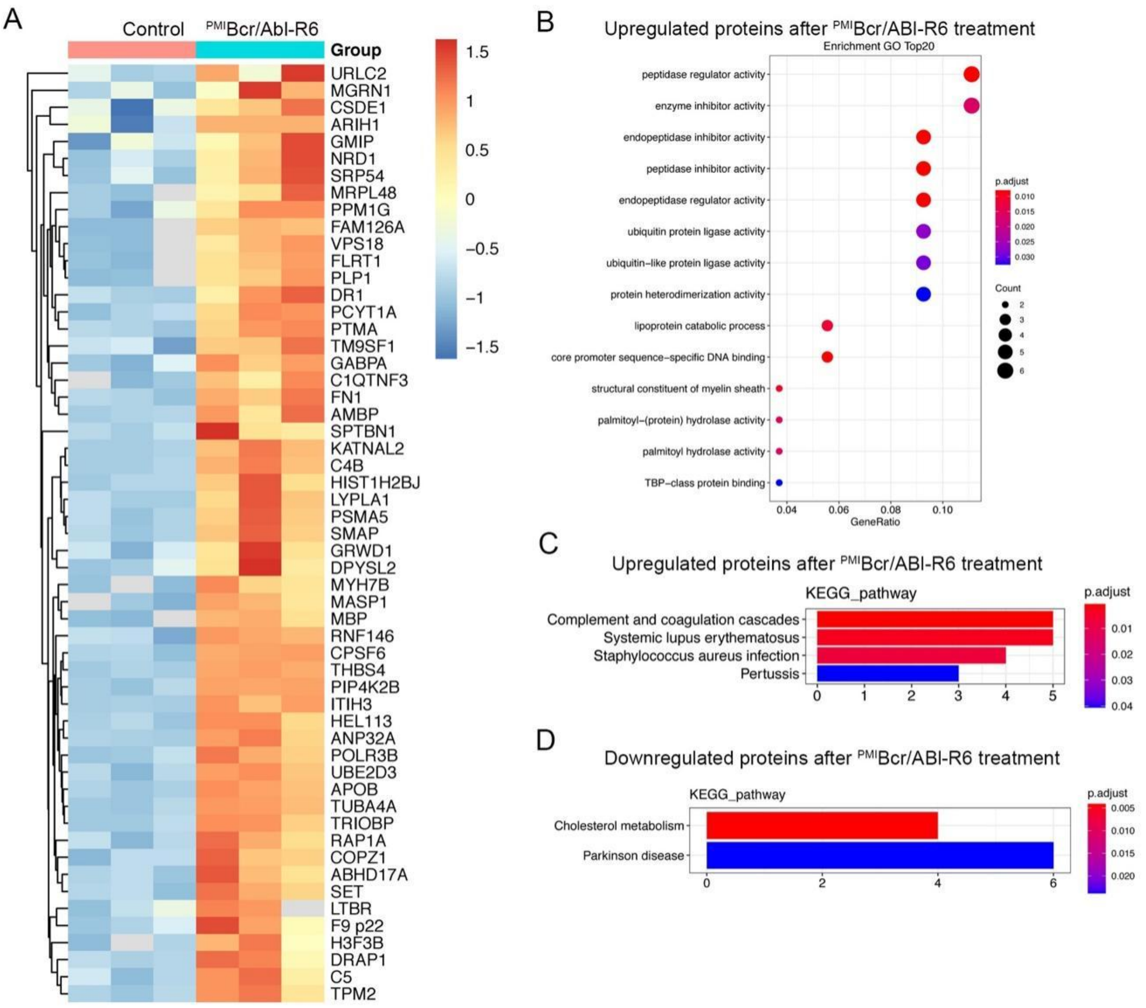

Figure S8. Proteomics analysis of KU-812 cells after ^{PMI}Bcr/Abl-R6 treatment. (A) Heat map analysis of up-regulated proteins in KU-812 cells after treatment with ^{PMI}Bcr/Abl-R6. (B) GO enrichment analysis of up-regulated signal pathways after ^{PMI}Bcr/Abl-R6 treatment in KU-812 cells. (C) KEGG pathway enrichment analysis of up-regulated proteins after ^{PMI}Bcr/Abl-R6 treatment in KU-812 cells. (D) KEGG pathway enrichment analysis of down regulated proteins after ^{PMI}Bcr/Abl-R6 treatment in KU-812 cells.

Fig. S9

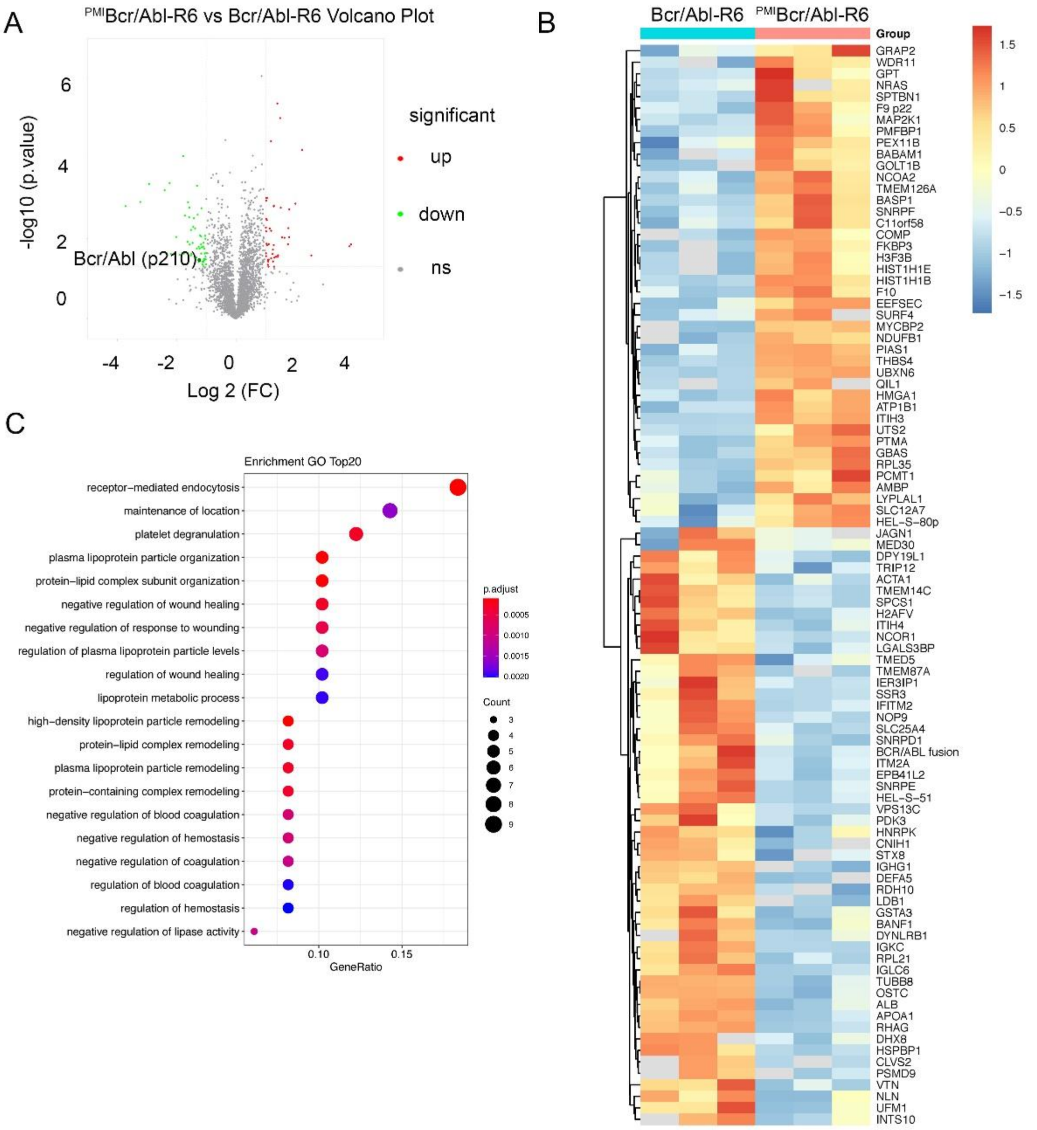

Figure S9. Proteomics analysis of KU-812 cells compared between Bcr/Abl-R6 and ^{PMI}Bcr/Abl-R6treatment. (A) Volcano plots of proteomics analysis in KU-812 cells compared between Bcr/Abl-R6 and ^{PMI}Bcr/Abl-R6 treatment. (B) Heat map analysis of proteomics analysis in KU-812 cells compared between Bcr/Abl-R6 and ^{PMI}Bcr/Abl-R6treatment. (C) GO enrichment analysis compared between Bcr/Abl-R6 and ^{PMI}Bcr/Abl-R6 treatment in KU-812 cells.

Fig. S10

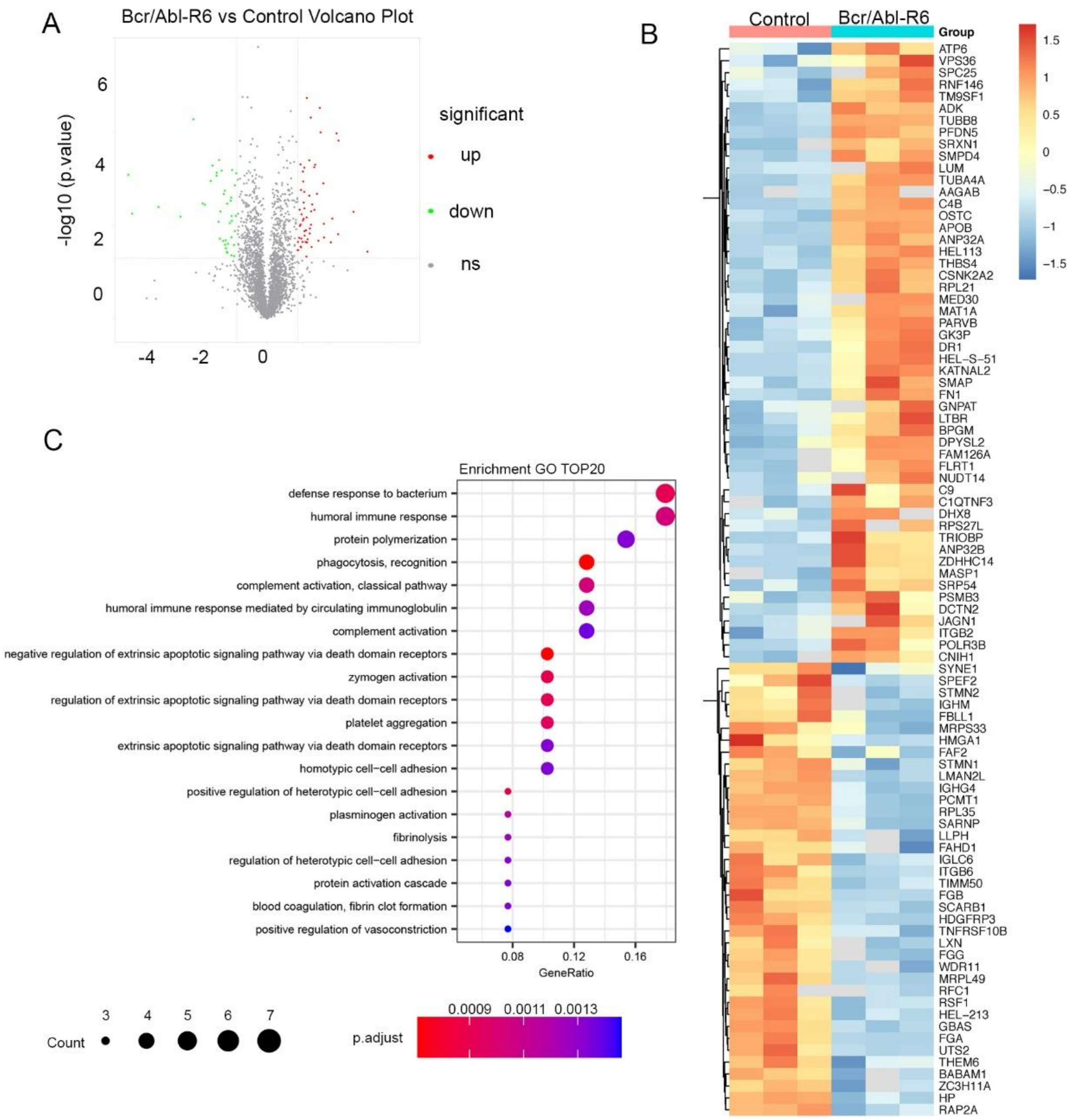

Figure S10. Proteomics analysis of KU-812 cells after Bcr/Abl-R6 treatment. (A) Volcano plots of proteomics analysis in KU-812 cells after treatment with Bcr/Abl-R6. (B) Heat map analysis of proteomics analysis in KU-812 cells after treatment with Bcr/Abl-R6. (C) GO enrichment analysis of Bcr/Abl-R6 treatment in KU-812 cells.

Figure S11

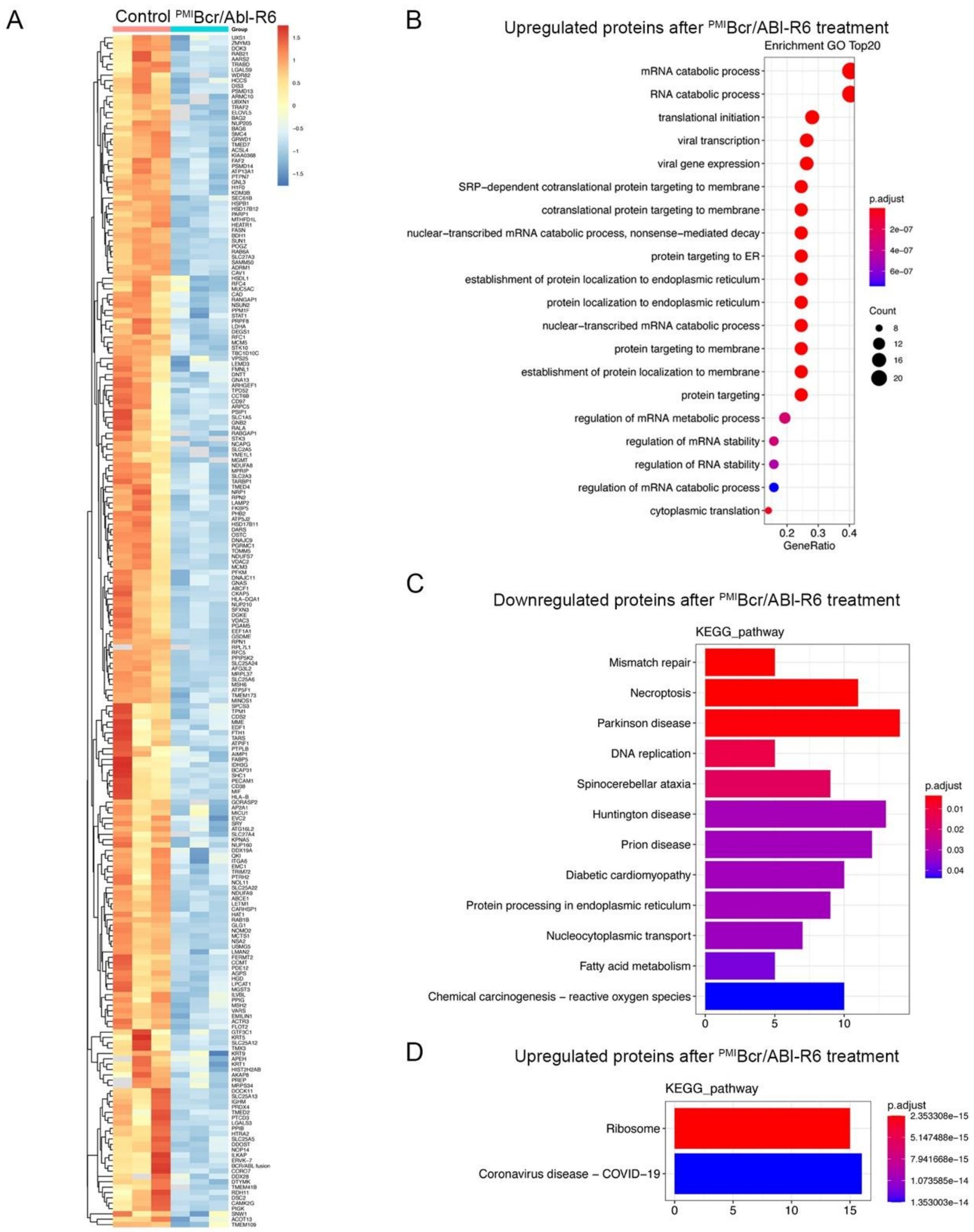

Figure S11. Proteomics analysis of SUP-B15 cells after ^{PMI}Bcr/Abl-R6 treatment. (A) Heat map analysis of downregulated proteins in SUP-B15 cells after treatment with ^{PMI}Bcr/Abl-R6. (B) GO pathways enrichment analysis of upregulated proteins after ^{PMI}Bcr/Abl-R6 treatment in SUP-B15 cells. (C) KEGG pathway enrichment analysis of downregulated proteins after ^{PMI}Bcr/Abl-R6 treatment in SUP-B15 cells. (D) KEGG pathway enrichment analysis of upregulated proteins after ^{PMI}Bcr/Abl-R6 treatment in SUP-B15 cells.

Figure S12

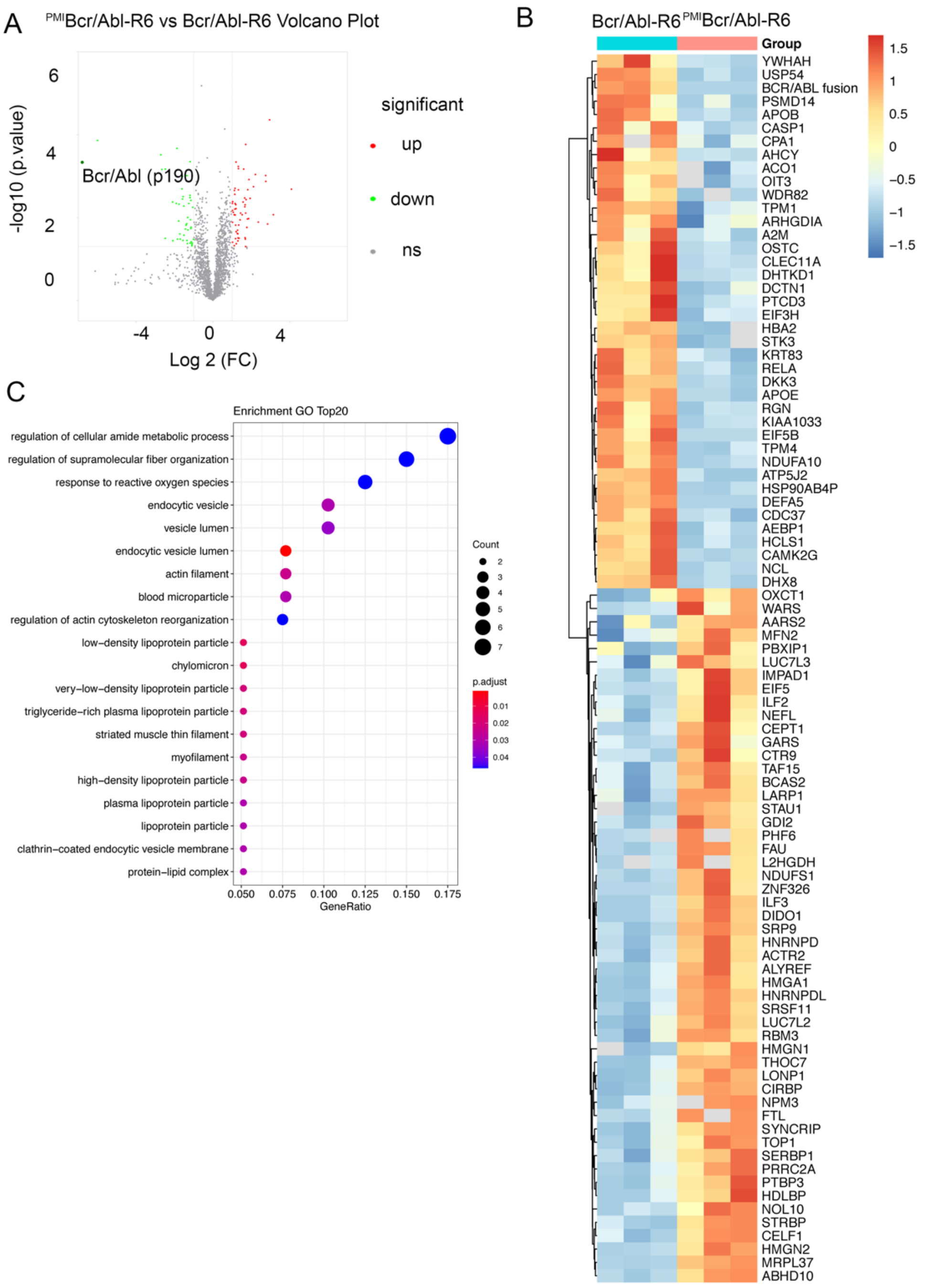

Figure S12. Proteomics analysis of SUP-B15 cells compared between Bcr/Abl-R6 and ^{PMI}Bcr/Abl-R6 treatment. (A) Volcano plots of proteomics analysis in SUP-B15 cells compared between Bcr/Abl-R6 and ^{PMI}Bcr/Abl-R6 treatment. (B) Heat map analysis of proteomics analysis in SUP-B15 cells compared between Bcr/Abl-R6 and ^{PMI}Bcr/Abl-R6treatment. (C) GO enrichment analysis compared between Bcr/Abl-R6 and ^{PMI}Bcr/Abl-R6treatment in SUP-B15 cells.

Figure S13

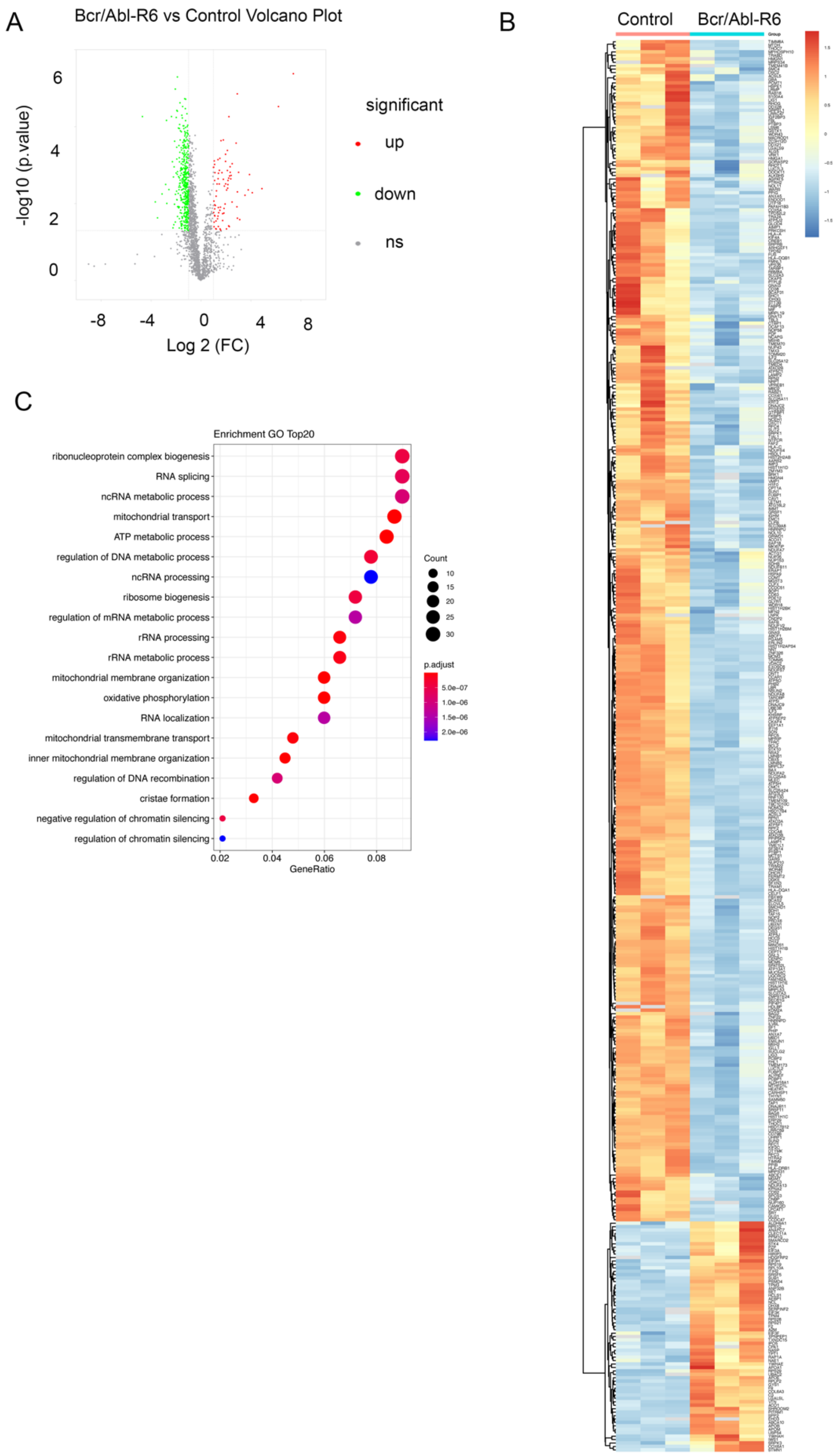

Figure S13. Proteomics analysis of SUP-B15 cells after Bcr/Abl-R6 treatment. (A) Volcano plots of proteomics analysis in SUP-B15 cells after treatment with Bcr/Abl-R6. (B) Heat map analysis of proteomics analysis in SUP-B15 cells after treatment with Bcr/Abl-R6.

(C) GO enrichment analysis of Bcr/Abl-R6 treatment in SUP-B15 cells.

Figure S14

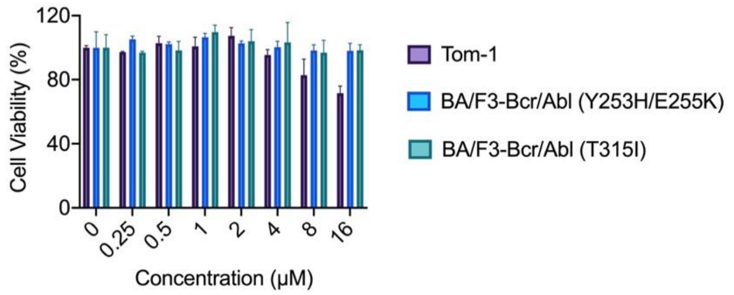

Figure S14. Cell viability of Tom-1, BA/F3-Bcr/Abl (Y253H/E255K) and BA/F3-Bcr/Abl (T315I) cells 24h after treatment with varying concentrations of imatinib.

Figure S15

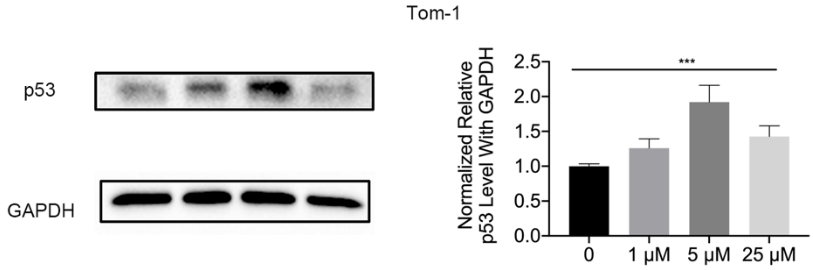

Figure S15. PMIBcr/Abl-R6 activate p53 in Tom-1 cells. Representative western blotting and analysis of p53 after treatment with PMIBcr/Abl-R6 at different concentrations and statistical analysis of data from three independent assays (n=3; ***indicates $p<0.001$.)

Figure S16

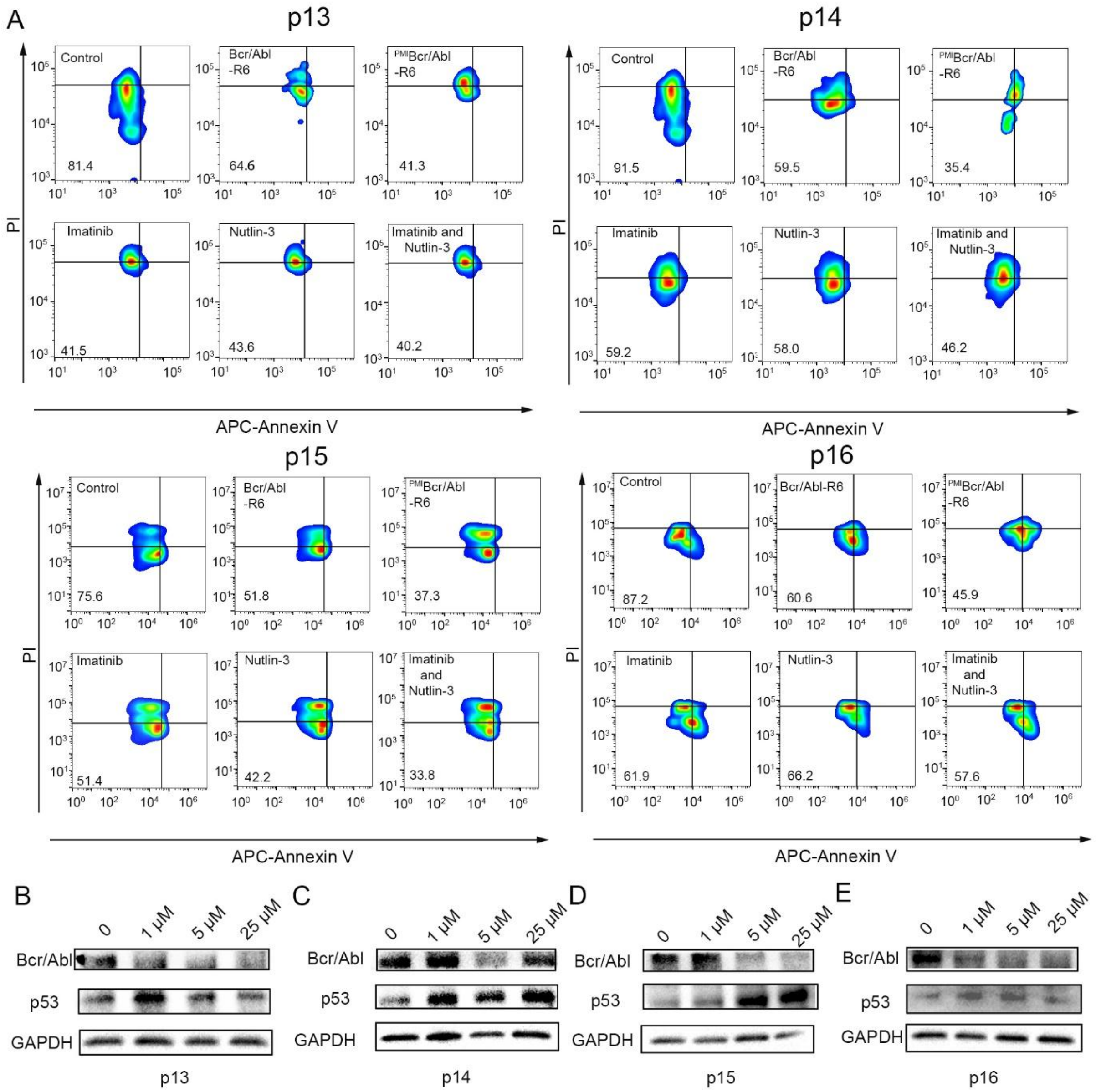

Figure S16. ^{PMI}Bcr/Abl-R6 induces Bcr/Abl degradation, activates p53, and induces apoptosis in primary cells from patients' bone marrow biopsy. (A) Apoptosis of CML samples after ^{PMI}Bcr/Abl-R6, Bcr/Abl-R6, imatinib or/and nutlin-3 treatment detected by flow cytometry. (B) Immunoblotting analysis of Bcr/Abl and p53 after treatment with ^{PMI}Bcr/Abl-R6 in primary cells from patients at different concentrations for 24 hours.

Figure S17

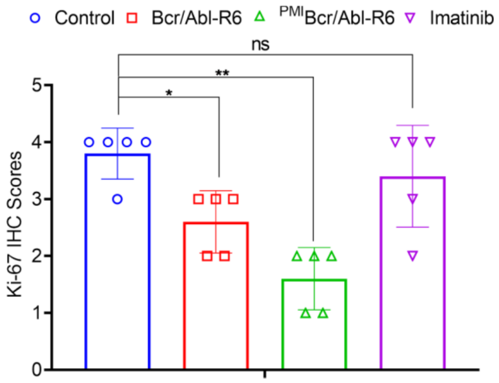

Figure S17. IHC analysis of Ki-67 in BA/F3-Bcr/Abl (T315I) xenograft tumors from each treatment group. IHC scores were determined with ImageJ: 4, highly positive; 3, positive; 2, minimally positive; 1, negative.All analysis for statistically significant differences were used T test. Statistical significance was represented by * stands for $p < 0.05$, ** stand for $p < 0.005$, ns stand for no significant difference.

Figure S18

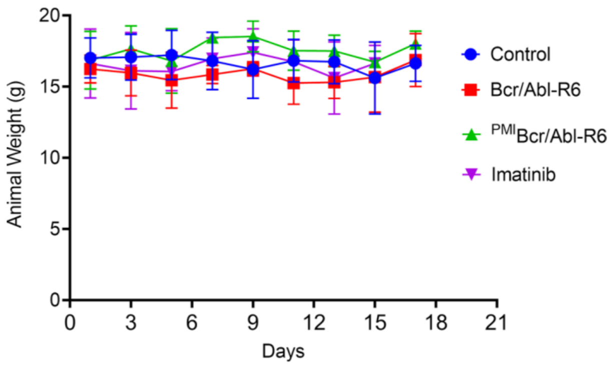

Figure S18. Animal body weight variation of different treatment groups.

Figure S19

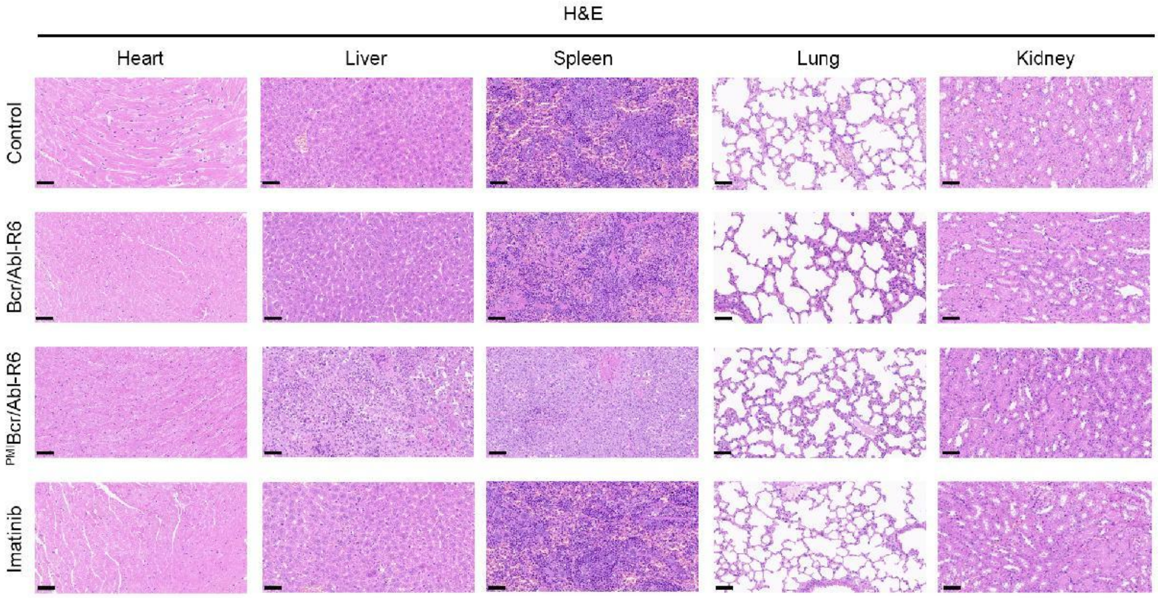

Figure S19. Representative histopathological analysis of organs from each treatment group using H&E staining assay (scale bar: 50 μm).

Figure S20

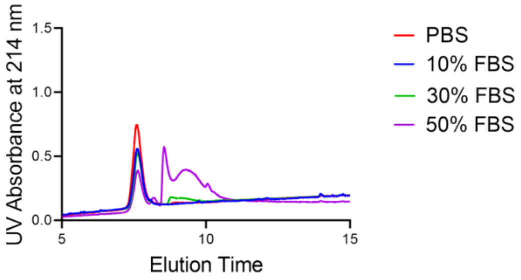

Figure S20. HPLC analysis of residual ^{PMI}Bcr/Abl-R6 after incubating with FBS.
